# Mitochondrial bioenergetic deficits in *C9orf72* amyotrophic lateral sclerosis motor neurons cause dysfunctional axonal homeostasis

**DOI:** 10.1007/s00401-020-02252-5

**Published:** 2021-01-04

**Authors:** Arpan R. Mehta, Jenna M. Gregory, Owen Dando, Roderick N. Carter, Karen Burr, Jyoti Nanda, David Story, Karina McDade, Colin Smith, Nicholas M. Morton, Don J. Mahad, Giles E. Hardingham, Siddharthan Chandran, Bhuvaneish T. Selvaraj

**Affiliations:** 1 UK Dementia Research Institute at University of Edinburgh, University of Edinburgh, Edinburgh bioQuarter, Chancellor’s Building, 49 Little France Crescent, Edinburgh, EH16 4SB UK; 2 Centre for Clinical Brain Sciences, University of Edinburgh, Edinburgh, UK; 3 Anne Rowling Regenerative Neurology Clinic, University of Edinburgh, Edinburgh, UK; 4 Euan MacDonald Centre for MND Research, University of Edinburgh, Edinburgh, UK; 5 Nuffield Department of Clinical Neurosciences, University of Oxford, Oxford, UK; 6 MRC Edinburgh Brain Bank, Academic Department of Neuropathology, University of Edinburgh, Edinburgh, UK; 7 Edinburgh Pathology, University of Edinburgh, Edinburgh, UK; 8 Centre for Discovery Brain Sciences, University of Edinburgh, Edinburgh, UK; 9 University/British Heart Foundation Centre for Cardiovascular Science, University of Edinburgh, Edinburgh, UK; 10 Centre for Brain Development and Repair, inStem, Bangalore, India

**Keywords:** Amyotrophic lateral sclerosis, Axon, Energy metabolism, Frontotemporal dementia, Mitochondria, Motor neuron, Neurodegeneration

## Abstract

Axonal dysfunction is a common phenotype in neurodegenerative disorders, including in amyotrophic lateral sclerosis (ALS), where the key pathological cell-type, the motor neuron (MN), has an axon extending up to a metre long. The maintenance of axonal function is a highly energy-demanding process, raising the question of whether MN cellular energetics is perturbed in ALS, and whether its recovery promotes axonal rescue. To address this, we undertook cellular and molecular interrogation of multiple patient-derived induced pluripotent stem cell lines and patient autopsy samples harbouring the most common ALS causing mutation, *C9orf72*. Using paired mutant and isogenic expansion-corrected controls, we show that *C9orf72* MNs have shorter axons, impaired fast axonal transport of mitochondrial cargo, and altered mitochondrial bioenergetic function. RNAseq revealed reduced gene expression of mitochondrially encoded electron transport chain transcripts, with neuropathological analysis of *C9orf72*-ALS post-mortem tissue importantly confirming selective dysregulation of the mitochondrially encoded transcripts in ventral horn spinal MNs, but not in corresponding dorsal horn sensory neurons, with findings reflected at the protein level. Mitochondrial DNA copy number was unaltered, both in vitro and in human post-mortem tissue. Genetic manipulation of mitochondrial biogenesis in *C9orf72* MNs corrected the bioenergetic deficit and also rescued the axonal length and transport phenotypes. Collectively, our data show that loss of mitochondrial function is a key mediator of axonal dysfunction in *C9orf72-*ALS, and that boosting MN bioenergetics is sufficient to restore axonal homeostasis, opening new potential therapeutic strategies for ALS that target mitochondrial function.

**Supplementary Information:**

The online version of this article (10.1007/s00401-020-02252-5) contains supplementary material, which is available to authorized users.

## Introduction

Amyotrophic lateral sclerosis (ALS) is an incurable, rapidly progressive and fatal neurodegenerative disorder, characterised by loss of upper and lower motor neurons (MNs) [18]. Humans are the only species affected by sporadic ALS [8]. Approximately 10–20% of ALS cases are familial, of which the *C9orf72* hexanucleotide repeat expansion mutation is the commonest cause [29, 81]. The finding that familial ALS is clinically and pathologically indistinguishable from sporadic ALS supports the study of monogenetic causes to better understand common pathogenic mechanisms [67]. Thus, human induced pluripotent stem cell (iPSC)-derived MN experimental platforms [84], combined with paired gene-edited isogenic control lines, provide a powerful approach, both to identify early-stage disease-driving mechanisms, and establish causality between a given mutation and phenotypes [31, 85, 91, 92]. Such in vitro disease modelling is complemented by in vivo approaches, particularly those that harness technologies interrogating molecular neuropathological signatures in human post-mortem tissue [40, 43, 92].

Although a clinical ALS phenotype that includes hypermetabolism and dyslipidaemia [32] is well recognised [103], there is limited understanding of the underlying mechanism and/or connection to MN metabolic state [13, 104, 109]. Neurons, in contrast to astrocytes (for example), are predominantly reliant upon mitochondrial oxidative phosphorylation [114]; this, coupled with the extraordinary length of MN axons—20,000 times longer than the diameter of their soma [47]—suggests a particular metabolic vulnerability of MNs to deficits in key processes involved in the maintenance of the form and function of the axon: dysfunctional axonal homeostasis. These processes include cytoskeletal dynamics and axonal transport. Both axonal growth, as well as transport of cargo driven by dynein and kinesin molecular motors, are energy demanding processes, with kinesin hydrolysing one ATP per 8 nm step [38]. Transport of mitochondrial cargo, in contrast to vesicular transport, depends on mitochondrial ATP, and not glycolysis [113]. Defective transport of cargo to the distal axon has been reported in several ALS causing mutations [1, 2, 9, 22, 28, 41, 48, 50, 64, 71, 78, 96], and not just in genes canonically involved in microtubule-based transport of cargo [70, 73, 98]. It is therefore perhaps surprising that, despite major advances in our understanding of the biology and consequence of the *C9orf72* repeat expansion mutation on MNs [10], its specific impact on axonal homeostasis has been relatively understudied, particularly in human models [1, 64, 96]. It remains, for example, unknown whether *C9orf72* MNs have a metabolic deficit, and whether this contributes to axonal dysfunction and selective MN vulnerability.

Against this background, we examined the cell-autonomous role of mitochondrial bioenergetics on *C9orf72* MN axonal function using multiple patient-derived iPSCs combined with isogenic controls, transcriptomic analysis and human neuropathological study. We establish that *C9orf72* MNs have dysfunctional axonal homeostasis, with aberrations in axonal morphology (reduced neurite length) and function (impaired fast axonal transport of mitochondrial cargo). We show that these axonal phenotypes are associated with concomitant metabolic dysfunction, owing to defective mitochondrial respiration. Unbiased RNA-sequencing revealed reduced expression of electron transport chain transcripts, encoded by mitochondrial DNA, the copy number of which was unaltered. Critically, we show, through neuropathological analysis of patient post-mortem tissue, that this transcriptomic dysregulation is selective to anterior horn spinal (motor) neurons, and is absent in dorsal horn spinal (sensory) neurons, with corresponding alterations reflected at the level of protein expression. Through manipulation of this molecular mitochondrial loss-of-function signature, leading to therapeutic rescue of the observed dysfunctional axonal homeostasis, we determine a novel causal relationship between axon dysfunction and contributory metabolic dysfunction in *C9orf72*-ALS.

## Materials and methods

### Cell culture and motor neuron generation from parental and gene-edited human iPSCs

Dermal fibroblasts from two unrelated healthy individuals (Con-1, Con-2) and three *C9orf72* ALS/FTD patients (C9-1, C9-2, C9-3) harbouring the G_4_C_2_ repeat expansion in the *C9orf72* gene were obtained and reprogrammed under full Ethical/Institutional Review Board approval at the University of Edinburgh, and isogenic controls were generated via CRISPR/Cas9 technology, as previously described by our group [92, 105] (Table 1). iPSCs were maintained in Matrigel^®^ Growth Factor Reduced Basement Membrane Matrix (354230, Corning^®^ Life Sciences)-coated plastic dishes in E8™ medium (A1517001, Gibco™ Thermo Fisher Scientific) at 37 °C and 5% CO_2_. Standard Giemsa banding chromosome analysis was periodically performed over the course of this study to exclude a karyotypic abnormality (The Doctors Laboratory Ltd, London), together with monthly testing of iPSC culture supernatants to exclude mycoplasma contamination using the Venor^®^GeM Classic detection kit (11-1050, Minerva Biolabs GmbH).

**Table 1:** Details of human iPSC control and *C9orf72* lines

Line	Reprogramming method	Repeat Length	Sex	Ethnicity	Age of onset (years)	Age at skin biopsy (years)	Disease duration (months)	Diagnosis	Family history	Site of onset	Dementia
Con-1	Retroviral	N/A	Female	Unknown	N/A	40	N/A	N/A		N/A	No
Con-2	Episomal	N/A	Female	Caucasian	N/A	56	N/A	N/A	Psychiatric disorders	N/A	No
C9-1	Sendai	*c*. 750	Female	Dutch	38	39	31	ALS/FTD (pm confirmed)	ALS/FTD	Behavioural change, language loss, wasting of hand muscles	Yes
C9-2	Retroviral	*c.* 638	Male	Caucasian	52	58	72	ALS	Mother-dementia	Lower limb	Cognitive decline
C9-3	Retroviral	*c.* 960	Male	Dutch	65	67	36	ALS	ALS/FTD	Lower limb	No

Spinal MN differentiation was performed using an established protocol [62] with minor modifications, yielding a highly enriched and electrophysiologically mature neuronal culture, devoid of glia, with *circa* 60% of cells being positive for islet-1 and islet-2 homeobox MN markers one week post platedown [92]. iPSCs were dissociated into single cells using 1X Accutase® (A6964, Sigma-Aldrich^®^), and neuralised as a suspension culture using dual-SMAD inhibition by SB-431542 (20 μM; 1614, Tocris^®^ Bio-Techne), LDN-193189 (0.1 μM; S2618, Selleckchem) and potentiation with the Wnt-agonist, CHIR-99021 (3 μM; 4423, Tocris^®^ Bio-Techne) in N2/B27 medium (0.5X Neurobasal™ [21103049, Gibco™ Thermo Fisher Scientific], 0.5X Advanced DMEM/F12 [12634028, Gibco™ Thermo Fisher Scientific], 1X Antibiotic–Antimycotic [15240062, Gibco™ Thermo Fisher Scientific], 1X GlutaMAX™ [35050061, Gibco™ Thermo Fisher Scientific], 100 μM beta-mercaptoethanol [31350010, Gibco™ Thermo Fisher Scientific], 1X B-27™ supplement [17504044, Gibco™ Thermo Fisher Scientific], 1X N-2 supplement [17502001, Gibco™ Thermo Fisher Scientific] and 10 μM l-ascorbic acid [A4403, Sigma-Aldrich^®^]). On day 2, neural spheres were simultaneously patterned to spinal cord identity by treating with retinoic acid (RA, 0.1 μM; R2625, Sigma-Aldrich^®^) and smoothened agonist (SAG, 0.5 μM; 566660, Sigma-Aldrich^®^), promoting caudalisation and ventralisation, respectively, along with SB-431542, LDN-193189 and CHIR-99021 in N2/B27 medium for an additional 5 days. On day 7, spheres were maintained in culture with RA and SAG, with the addition of recombinant human brain-derived neurotrophic factor (BDNF, 10 ng/ml; 248-BDB, R & D Systems^®^ Bio-Techne) in N2/B27 medium to generate MN progenitors. From day 9, MN progenitors were cultured in day 7 medium with the addition of DAPT (10 μM; 2634, Tocris^®^ Bio-Techne), an inhibitor of Notch signalling, for an additional 5–7 days. At day 14–16, MN spheres were dissociated using 0.05% Trypsin–EDTA (25300054, Gibco™ Thermo Fisher Scientific) and cells were plated as a monolayer onto laminin from Engelbreth-Holm-Swarm murine sarcoma basement membrane (5 μg/ml; L2020, Sigma-Aldrich^®^), fibronectin human plasma (10 μg/ml; F2006, Sigma-Aldrich^®^), and Matrigel^®^ (1:20)-coated dishes that had been pre-treated with poly-l-ornithine bromide (100 µg/ml; P3655, Sigma-Aldrich^®^). Dissociated MNs were cultured in motor neuron neurotrophic factor (MN-NF) medium (1X Neurobasal, 1X Antibiotic–Antimycotic, 1X GlutaMAX™, 1X MEM Non-Essential Amino Acids solution [11140–035, Gibco™ Thermo Fisher Scientific], 100 μM beta-mercaptoethanol, 1X B-27™ supplement, 1X N-2 supplement, RA [1 μM], ascorbic acid [2.5 μM], BDNF [10 ng/ml], recombinant human glial-derived neurotrophic factor [GDNF, 10 ng/ml; 212-GD, R & D Systems^®^ Bio-Techne], recombinant human ciliary neurotrophic factor [CNTF, 10 ng/ml; 257-NT, R & D Systems^®^ Bio-Techne], and animal-free recombinant human insulin-like growth factor-1 [IGF-1, 10 ng/ml; AF-100–11, PeproTech^®^]). MN-NF medium was regularly changed every 2–3 days and supplemented with uridine/5-fluoro-2′-deoxyuridine (1 μM, U/FDU; U3003 and F0503, Sigma-Aldrich^®^) for at least 1 week post platedown to remove residual proliferating cells.

### Overexpression of PGC1α

Briefly, FLAG-PGC1α-6xHIS (originated from Addgene [67637]) and P2A-eGFP (originated from pDONR-P2A-eGFP), were shuttled into a lentiviral backbone pLenti6-cppt-delta CMV-DEST-opre, to generate a FLAG-PGC1α-6xHIS-P2A-eGFP lentiviral construct. PGC1α overexpression lentivirus was generated as previously described [63, 76] and transduced as previously described by our group [56] in human iPSC-derived MNs at a multiplicity of infection (MOI) of 10. This achieved a mean transduction efficiency of *circa* 70% MNs. Vehicle control transduction was achieved by lentiviral transduction of emerald GFP in the equivalent plasmid backbone using a plasmid from Invitrogen™ (Thermo Fisher Scientific, V35520).

### Immunocytochemistry and neurite outgrowth quantification

MNs plated on 30-mm glass coverslips etched by the pre-treatment of nitric acid were washed once with phosphate-buffered saline (PBS; 10010, Gibco™ Thermo Fisher Scientific) and then incubated for 20 min at room temperature with 4% paraformaldehyde (AGR1026, Agar Scientific). Thereafter, cells were washed three times with PBS, followed by incubation for 10 min at room temperature with PBS containing 0.2% Triton X-100 (T8787, Sigma-Aldrich^®^) for permeabilisation, followed by a 1 h incubation at room temperature in 6% normal goat serum blocking buffer (S-1000, Vector Laboratories) in PBS. Primary antibodies diluted in blocking buffer were then added for overnight incubation at 4 °C. Cells were washed three times in phosphate-buffered saline plus 0.1% Tween^®^ 20 (PBST; P9416, Sigma-Aldrich^®^) before the secondary antibodies coupled to fluorophores (Alexa Fluor^®^ range, Thermo Fisher Scientific) were added diluted in PBST for 1 h at room temperature, with the samples protected from light. After washing with PBST, 4′,6-diamidino-2-phenylindole (DAPI, 1 µg/ml diluted in PBS; 10236276001, Roche) was applied for nuclear counterstaining for a further 15 min. Coverslips were then washed with PBST and distilled water, and then mounted onto glass slides (12362098, Thermo Fisher Scientific) with FluorSave™ Reagent (345789, Millipore) and imaged using an Axio Observer Z1 inverted motorised microscope (Carl Zeiss). Images were acquired from random positions of the coverslip. Primary antibodies used in this study are as follows: SMI-312 at 1:1000 (837904, BioLegend^®^), GFP at 1:1000 (ab13970, Abcam), and PGC1α at 1:300 (ab54481, Abcam). Neurite outgrowth was quantified 7 days post platedown (plating density of 5000 MNs per coverslip) blinded to sample genotype using the Simple Neurite Tracer plugin [58] within Fiji [89].

### RNA extraction, RNA sequencing and transcriptomic analysis

Total RNA was extracted from MNs from two independent isogenic-corrected paired cell lines and two independent control cell lines (C9-1 and C9-2; Con-1 and Con-2) at day 21 post platedown using the RNeasy^®^ Mini kit (74104, Qiagen^®^), according to the manufacturer’s instructions. RNA samples were assessed for concentration (NanoDrop ND-100 Spectrometer, NanoDrop Technologies) and quality (Agilent 2200 TapeStation, Agilent Technologies) before library preparation. Library preparation and sequencing were carried out by Edinburgh Genomics (Edinburgh, UK). For each sample, cDNA was converted to a sequencing library using the TruSeq stranded mRNA-seq library. Barcoded libraries were pooled and sequenced on an Illumina HiSeq 4000 using 75 base paired-end reads to generate at least 46 million raw reads per sample. The reads were mapped to the primary assembly of the human (hg38) reference genome contained in Ensembl release 90 [25]. Alignment was performed with STAR version 2.5.3a [30]. Tables of per-gene read counts were generated from the mapped reads with featureCounts version 1.5.2 [55]. Differential gene expression analysis, using DESeq2 version 1.16.1, specifically examined the intersection in commonly and concordantly differentially expressed genes between the two mutant-isogene pairs, using a false discovery rate of 20%, achieved by setting a Benjamini–Hochberg corrected *p* value threshold of 0.2 (genes with an average Fragments Per Kilobase of transcript per Million mapped reads [FPKM] < 1 were disregarded). Gene ontology (GO) [5, 100] analysis was performed on the differentially expressed genes to identify putatively altered pathways or processes using the GeneRanker module [11] within Genomatix proprietary software suite (Intrexon Bioinformatics Germany GmbH; threshold: *p* ≤ 0.01, Fisher’s exact test). Competitive gene set testing was performed using CAMERA [110]. Promoter analysis was performed using default parameters within Genomatix; first, the Gene2Promoter module was used to extract promoter sequences of all significantly dysregulated genes annotated in hg38. Overrepresented transcription factor binding sites (TFBS) were searched using MatBase within the input regions, and statistics on TFBSs were generated, together with overrepresentation values and *Z*-scores (the distance from the population mean in units of the population standard deviation) compared against genomic and promoter background. Promoter associations were defined as transcription factor families known to occur more than twice as often in promoters as in genomic sequence. A *Z*-score below -2 or above 2 can be considered statistically significant (it corresponds to a *p* value of ~ 0.05).

### Quantitative reverse transcription PCR

Total RNA was extracted using the RNeasy^®^ Mini kit (74104, Qiagen^®^), according to the manufacturer’s instructions. Genomic DNA was removed using the on-column DNase method (79254, Qiagen^®^). cDNA was synthesised using 250 ng of RNA using the DyNAmo™ cDNA Synthesis Kit (10748908, Thermo Fisher Scientific). Technical replicates, as well as no template and no RT negative controls, were included, and at least three biological replicates were studied in each case. Real-time reverse transcription quantitative PCR (RT-PCR) reactions were set up with DyNAmo ColorFlash SYBR Green qPCR kit (10442308, Thermo Fisher Scientific) and run on a CFX96 System (Bio-Rad). The data were visualised and exported using Maestro 1.1 software (Bio-Rad). Primers were synthesised by Sigma-Aldrich^®^. Primer sequences and annealing temperatures used in this study are provided in Supplementary Table 1, online resource.

### Assessment of in vitro mitochondrial copy number

MNs, plated at a density of 2–4 million cells per 60 mm dish (430166, Corning^®^ Life Sciences), were lysed at day 14 post platedown using 200 µl of Bradley Lysis buffer (10 mM Tris–HCl pH 7.5, 10 mM EDTA pH 8.0, 0.5% w/v SDS, 10 mM NaCl) containing 0.2 mg/ml of fresh Proteinase K (AM2546, Ambion™) and incubated in locking-lid microfuge tubes at 55 °C overnight on a ThermoMixer^®^ comfort (Eppendorf AG) [80]. Subsequent steps are derived from classical techniques for genomic DNA extraction using phenol-choloroform [83]. An equal volume of phenol/choloroform/isoamyl alcohol solution (25:24:1; P2069, Sigma-Aldrich^®^) was added to each sample. After vortexing, organic and aqueous phases were separated by centrifugation at 16,000 rcf for 5 min. The upper aqueous phase was transferred to a new microfuge tube, and 200 µl of elution buffer (pH 8; 11–05-01–13, Integrated DNA Technologies, Inc) was added the original tube, which was vortexed and centrifuged as previously. The upper aqueous phase was transferred and combined with that obtained previously. An equal volume of chloroform/isoamyl alcohol solution (24:1; 25666, Sigma-Aldrich^®^) was added (choloroform back-extraction step). Following vortexing and centrifugation, the upper aqueous phase was transferred into a new microfuge tube, to which ammonium acetate (A2706, Sigma-Aldrich^®^) was added to a final concentration of 0.75 M, together with 20 µg of glycogen (9510, Ambion™). After mixing well, 2.5X volume of 100% ethanol was added. After a short 20 °C incubation on a ThermoMixer^®^, the samples were centrifuged at 4 °C at 16,000 rcf for 20 min. The supernatant was carefully decanted, and the pellet washed with 300 µl of cool 80% ethanol. After vortexing the samples, they were centrifuged at 16,000 rcf for 15 min at 4 °C. The supernatant was again carefully decanted and the pellet washed for a second time with 80% ethanol, followed by vortexing and centrifugation. The ethanolic solution was carefully aspirated and the pellet air dried at room temperature for 5 min followed by resuspension of the pellet in elution buffer, with gentle rocking overnight at 4 °C. Samples were assessed for concentration and quality using a NanoDrop ND-100 Spectrometer and stored at 4 °C for the duration of the study.

Mitochondrial DNA content was assessed by quantification of a unique (and rarely deleted) mitochondrial fragment in the minor arc of the mitochondrial genome (MT-ND1) relative to a single copy region of a nuclear gene (beta-2 microglobulin, B2M) using an established method [19, 39, 44, 61]. First, a customised double-stranded ND1–beta-2 microglobulin DNA fragment of 255 base pairs was synthesised as a gBlocks™ Gene Fragment (Integrated DNA Technologies, Inc.; sequence: 5′-TGC ATG ATC TAC GTG CGT CAC ATG CAG TAC CCA GCA GAG AAT GGA AAG TCA AAT TTC CTG AAT TGC TAT GTG TCT GGG TTT CAT CCA TCC GAC ATT GAA GTT GAC TTA CTG AAG AAT GGA GAG AGA CAC TAG CTC AGA TTC AGT AGA CCG CTG TTG CCC TAA AAC CCG CCA CAT CTA CCA TCA CCC TCT ACA TCA CCG CCC CGA CCT TAG CTC TCA CCA TCG CTC TAG TAA TGC AGA CAC TTG CGG TCC ATC TCG-3′). Serial dilutions were generated at 1:10 increments between 1 × 10^–8^ ng/µl and 10 ng/µl and stored at 4 °C for the duration of the study. Copy number was calculated as follows:

$$C \times M \times 1 \times {10}^{-15} \, {\text{mol/fmol}} \times \text{Avogadro{'}s\,number}=\text{copy number/} {\upmu}{\text{l}},$$

where *C* is the concentration of the gBlocks™ Gene Fragment in ng/µl, and *M* is the molecular weight (specified as 6.35 fmoles/ng).

A standard curve was generated. Real-time quantitative PCR (qPCR) reactions of the standards and the samples were set up in triplicate per experiment with DyNAmo ColorFlash SYBR Green qPCR kit (10442308, Thermo Fisher Scientific) and run on a CFX96 System (Bio-Rad). The data were visualised and exported using Maestro 1.1 software (Bio-Rad). Primers were synthesised by Sigma-Aldrich^®^. Primer sequences for ND1 and beta-2 microglobulin, and their annealing temperatures, are provided in Supplementary Table 1, online resource. To ensure the sample quantification cycles (Cq) for both reactions were within an optimal detection range of 17–33 [39], sample DNA concentrations differed by an order of magnitude between the mitochondrial (0.02X) and nuclear (1X) reactions. A standard curve of mean Cq value (ordinate) *versus* copy number (abscissa) was generated for both ND2 and beta-2 microglobulin, and copy numbers for each sample were interpolated using the semi-log line least squares fit function within Prism version 9.0.0 (GraphPad Software). The interpolated mitochondrial copy number was divided by the interpolated nuclear copy number to generate ratios of normalised values. Each experiment was performed at least three times (i.e., from at least three MN differentiations per cell line).

### Western blot

MNs were cultured for 3 weeks. MN-NF medium was removed and cells were washed with ice-cold PBS. MNs were lysed with ice-cold RIPA buffer (150 mM NaCl, 50 mM Tris–HCl, 2 mM EDTA, 1% Triton X-100, 0.1% SDS, 0.5% sodium deoxycholate, pH 7.4; all constituents from Sigma-Aldrich^®^) supplemented with 1X cOmplete EDTA-free protease and phosphatase (PhosSTOP) inhibitor cocktail tablets (04693159001, 04906837001, Roche Diagnostics). MNs were gently scraped off the dish and samples were sonicated for 3 × 5 s at amplitude 60% using a Branson Digital Sonifier 450 (Branson Ultrasonics), keeping them on ice in between pulses. Samples were then centrifuged at 16,000 rcf at 4 °C for 10 min and the supernatant was retained for protein quantification using a bicinchoninic acid assay (23225, Pierce™ Thermo Fisher Scientific). 20 µg of protein was added to Laemmli buffer [51], whence samples were incubated at 37 °C for 30 min prior to being loaded onto a Novex™ Tris–glycine gel (XP00145BOX, Invitrogen™) and blotted to a polyvinylidene difluoride (PVDF) Immobilon^®^-FL membrane (05,317, Millipore) using 10 mM 3-(Cyclohexylamino)-1-propanesulfonic acid (CAPS) buffer pH 11 (C2632, Sigma-Aldrich^®^) plus 10% methanol at 4 °C using a 150 mA transfer for 2 h. Total protein was quantified using Revert™ 700 Total Protein Stain (926–11,015, LI-COR Biosciences) as per manufacturer instructions, followed by blocking with Odyssey^®^ blocking buffer in PBS (927–40,000, LI-COR Biosciences) for 1 h at room temperature. Thereafter, primary antibody incubation in blocking buffer plus 0.2% Tween^®^ 20 (P9416, Sigma-Aldrich^®^) occurred at 4 °C overnight. The membrane was then washed with PBST three times and LI-COR IRDye^®^ infrared dyes (1:15,000) added to blocking buffer plus 0.2% Tween^®^ 20 and 0.01% SDS (L3771, Sigma-Aldrich^®^) for 1 h at room temperature. The membrane was then washed in PBST and imaged using an Odyssey CLx (LI-COR Biosciences). Blots were analysed using Empiria Studio 1.1 (LI-COR Biosciences). Primary antibodies used in this study are: Total OXPHOS Human WB Antibody Cocktail 1:1000 (ab110411, Abcam), VDAC1/Porin 1:2000 (ab15895, Abcam), and GAPDH 1:5000 (CB1001, Sigma-Aldrich^®^).

### Axonal transport studies

MNs, plated on laminin/Matrigel^®^/fibronectin-coated µ-slide 8-well ibidi dishes (80826, ibidi GmbH) at a density of 50,000–70,000 cells per well were sparsely transduced with lentivirus expressing mitoDsRed2 at platedown using an MOI of 0.5, optimised to visualise 1–2 labelled cells per field of view, as previously described by our group [37, 56]. Fluorescence live-cell imaging of mitochondrial axonal transport was performed at day 21 post platedown at 63X magnification (Plan-Apochrat 1.40 NA oil DIC M27 objective, Carl Zeiss) using an Axio Observer Z1 inverted motorised microscope (Carl Zeiss) equipped with a Cy3 FL filter-set (Carl Zeiss), Zen 2011 z-stack, time lapse and Definite Focus modules (Carl Zeiss), and an S1 Environmental System (Carl Zeiss) incubation chamber maintained at 37 °C and 5% CO_2_. Medium was changed 30 min prior to imaging to phenol red-free Neurobasal™ (12348017, Gibco™ Thermo Fisher Scientific) supplemented with 1X GlutaMAX™. Mitochondrial motility was recorded for 5 min using a 0.2 Hz capture of a ~ 100 µm stretch of axon and a small z-stack. ‘Proximal’ axon measurements were acquired from the axon immediately adjoining the cell body, and ‘distal’ were measured from the distal tip. At least four axons (*n*) were imaged per line per differentiation (*N*, where *N* = 3). MN genotype was counterbalanced and interleaved between experimental runs. Maximum intensity projections were computed in Fiji. Kymographs were generated and analysed using KymoToolBox [113] in Fiji [89] to determine the numbers of stationary (≤ 0.1 µm/s) *versus* bidirectionally motile mitochondria (either predominantly towards or away from the soma).

### Metabolic profiling

MNs were plated on polyethyleinimine (2.2 mg/ml in 0.1 M borate buffer, pH 8.4; 408727 and B3545, Sigma-Aldrich^®^)-treated V28 Seahorse plates (100882-004, Agilent), that had been coated overnight with laminin/Matrigel^®^/fibronectin as described above, at a high density (200,000 cells per well) to ensure the presence of a confluent cellular monolayer. A modification of the standard Agilent Seahorse XF Cell Mito Stress Test protocol was performed at day 14 post platedown using a Seahorse XFe24 Analyzer (Agilent), providing a complete mitochondrial bioenergetic profile, revealing critical information not evident in basal metabolism measurements alone. Cells were transitioned from the MN-NF medium to low-buffering capacity Seahorse XF Base Medium (102,353, Agilent) supplemented with 1X Glutamax™, 10 mM glucose (Sigma-Aldrich^®^) and 2 mM pyruvate (Sigma-Aldrich^®^), pH 7.35 ± 0.5 at 37 °C and maintained in ambient CO_2_ for 30 min prior to the plate being inserted into the machine. Oxygen consumption rate (OCR), an indicator of oxidative phosphorylation and therefore mitochondrial respiration, and extracellular acidification rate (ECAR), an indicator of lactate export and anaerobic glycolysis, were measured during sequential injection of optimised concentrations of oligomycin (3 μM; Sigma-Aldrich^®^), carbonyl cyanide 4-(trifluoromethoxy)phenylhydrazone (FCCP; Cayman Chemical, Cambridge Bioscience), antimycin/rotenone (3 μM; Sigma-Aldrich^®^), and 2-deoxy-d-glucose (1 M; D6134, Sigma-Aldrich^®^). FCCP dose was optimised for each cell line (low: 0.125 μM; medium: 0.25 μM; high: 0.5 μM) and the concentration adopted that maximised the OCR response. The protocol adopted a 3 min mixing, 2 min wait, 3 min measure cycle (i.e., cycle total duration of 8 min). Three measurements were taken basally (i.e., three cycles with a total duration of 24 min), and three measurements were taken after injection of each drug (in sequence: oligomycin for inhibiting ATP-linked respiration, FCCP for eliciting maximal uncoupled respiration, antimycin/rotenone for inhibiting the electron transport chain, and 2-deoxy-d-glucose for terminating glycolysis; see Supplementary Fig. 2a, 2b, online resource). The measured OCR was normalised to total protein [23] using the bicinchoninic acid assay (23225, Pierce™ Thermo Fisher Scientific). A minimum of four individual wells (*n* ≥ 4) per line were included per plate, with experiments repeated in three independent cultures from different differentiations (*N* = 3). Data were visualised in, and imported from, Seahorse Wave Desktop software (version 2.6.0.31, Agilent). Basal respiration was calculated by subtracting the minimum OCR value after antimycin/rotenone injection (measurement cycle 12) from the OCR value prior to injection of oligomycin (measurement cycle 3). FCCP-stimulated maximal uncoupled respiration was calculated by subtracting the minimum OCR after antimycin/rotenone injection (measurement cycle 12) from the maximum OCR after FCCP injection (measurement cycle 7). Basal glycolytic rate was estimated by subtracting the ECAR value immediately after injection of 2-deoxy-d-glucose (measurement cycle 13) from the ECAR value immediately prior to the addition of oligomycin (measurement cycle 3). Respiratory-inhibited maximal glycolysis was inferred by subtracting the ECAR value immediately after the injection of 2-deoxy-d-glucose (measurement cycle 13) from the ECAR value immediately prior to the injection of 2-deoxy-d-glucose (measurement cycle 12).

### Post-mortem case identification

We identified a cohort of five *C9orf72*-ALS cases (from the Medical Research Council Edinburgh Brain Bank; Table 2) and five age- and sex-matched control cases (from the Sudden Death Brain Bank, with no neurological disorder during life and no significant neuropathology present at post-mortem; Supplementary Table 2, online resource) for neuropathological assessment of ventral and dorsal spinal cord, as adopted in our previously published work [40, 65, 92]. All clinical data were collected as part of Scottish Motor Neurone Disease Register (SMNDR) and Care Audit Research and Evaluation for Motor Neurone Disease (CARE-MND) platform (ethics approval from Scotland A Research Ethics Committee 10/MRE00/78 and 15/SS/0216). Additionally, all cases had corresponding whole-genome sequencing and diagnostic repeat prime PCR, demonstrating pathogenic repeat lengths in the *C9orf72* locus [53]. The use of human tissue for post-mortem studies has been reviewed and approved by the Edinburgh Brain Bank ethics committee and the Academic and Clinical Central Office for Research and Development medical research ethics committee, in line with the Human Tissue (Scotland) Act 2006.

**Table 2:** Clinical meta-data for *C9orf72* cases used in post-mortem work

Case ID (sex)	Age at death (years)	Disease duration (months)	Regions affected at death	Classification based on El Escorial criteria[60]	Past medical history	Smoker	Alcohol intake	Prescribed riluzole
Case 1 (F)	63	25	UL and LL	Amyotrophic lateral sclerosis (definite)	Migraine	Ex	Yes	No
Case 2 (M)	50	29	UL, LL and bulbar	Amyotrophic lateral sclerosis (probable)	Sarcoidosis	Ex	No	Yes
Case 3 (F)	62	37	UL, LL and bulbar	Amyotrophic lateral sclerosis (definite)	Lumbar discectomy	Yes	Yes	Yes
Case 4 (F)	62	50	UL, LL and bulbar	Amyotrophic lateral sclerosis (definite)	Nil known	No	Yes	No
Case 5 (M)	58	87	UL and LL	Amyotrophic lateral sclerosis (definite)	Lower back pain	Yes	Yes	Yes

*UL* upper limb, *LL* lower limb, *F* female, *M* male

### BaseScope™ RNA in situ hybridisation

Formalin-fixed paraffin embedded human post-mortem spinal cord tissue was sectioned at 4 µm thickness onto SuperFrost Plus™ slides (10149870, Fisher Scientific, Thermo Fisher Scientific) as previously reported [40], and BaseScope™ reagents (Advanced Cell Diagnostics™ Bio-Techne) were used as per manufacturer’s guidelines [106]. In brief, following deparaffinisation, tissue sections were incubated with hydrogen peroxide for 10 min at room temperature and target antigen retrieval was performed by submerging slides in BaseScope™ 1X target retrieval reagent at 99 °C in a Braun Multiquick FS 20 steamer for 15 min. The tissue was then permeabilised using BaseScope™ protease III at 40 °C for 30 min. Probe hybridisation was then performed by incubating the slides with four drops of custom-designed BaseScope™ probe: either BA-Hs-MT-ND2-3zz-st (853271, Advanced Cell Diagnostics™ Bio-Techne) or BA-Hs-MT-CO3-1zz-st (717961, Advanced Cell Diagnostics™ Bio-Techne), to recognise ND2 and CO3 mRNA transcripts, respectively, or negative control (l-2,3-dihydrodipicolinate reductase, DapB) or positive control (peptidyl-prolyl *cis*–*trans* isomerase, PPIB) probe for 2 hours at 40 °C. Following successive probe amplification steps, transcripts were detected using the BaseScope™ RED detection kit (322910, Advanced Cell Diagnostics™ Bio-Techne) and slides were counterstained using haematoxylin and lithium carbonate. The slides were subsequently cleared in xylene and mounted on a 24 × 50 mm coverslip using two drops of VectaMount^®^ mounting medium. Sections were then imaged at 20 × magnification on a NanoZoomer slide scanner (Hamamatsu), and the relative neuronal abundance of transcripts (denoted by counting the number of spots per cell) was assessed by neuropathologists, blinded to the demographic and clinical metadata underlying each sample. Motor and sensory neurons were identified based on their anatomical location within the spinal cord, and according to established neuropathological criteria, including size and morphology.

### BaseScope™ DNA in situ hybridisation

To examine for mitochondrial DNA copy number changes in post-mortem spinal cord neurons, we adapted a recently published, validated technique [21], permitting the application of BaseScope™ to achieve cell-type specific quantification. We optimised the BaseScope™ technique on control and diseased tissue to address probe specificity; thus, DNase treatment (optimised specifically to retain haematoxylin counterstaining, but eliminate probe binding, confirming that the probes were binding to DNA, and not RNA) and RNase treatment (optimised specifically to assess the extent of background probe binding to RNA, confirming that probes were binding to DNA, and not RNA) was carried out at 40 °C at a concentration of 800U/ml for DNase and RNase for 30 min immediately following the protease III tissue permeabilisation step, and prior to probe hybridisation. Probe hybridisation was then performed by incubating the slides with four drops of custom-designed BaseScope™ probe (BA-Hs-MT-CO1-3zz-st-sense; 891111, Advanced Cell Diagnostics™ Bio-Techne), targeting the antisense region of the *MT-CO1* gene (i.e., the reverse-complement sequence of 273–402 of NC_012920.1:5904–7445) to bind to DNA, for 2 h at 40 °C. The method [21] elegantly profits on the fact that, although transcription of the circular mitochondrial DNA is bidirectional, meaning that theoretically probes could hybridise to either DNA or RNA (with resultant ambiguity in signal interpretation), most light strand transcripts are rapidly degraded, including those antisense to the *MT-CO1* gene [12, 68]. The Fast RED chromogen incubation step was reduced from 10 to 8 min. Sections were imaged, anonymised, at 20 × magnification using a NanoZoomer slide scanner (Hamamatsu). Motor and sensory neurons were identified based on their anatomical location within the spinal cord and according to established neuropathological criteria, including size and morphology. The relative abundance of spots (denoting DNA) was quantified by neuropathologists, blinded to the demographic and clinical metadata underlying each sample, assessing the mean (± standard error) number of spots per cell across ten cells per condition. Notably, assuming that each motor neuron is *circa* 50 µm in maximum dimension, and that the photomicrograph captures only one 4 µm *z*-plane (range of spots per cell per 4 µm *z*-plane is between 1 and 8 dots), we anticipate that the average spots per cell equates to between 50 and 400 spots per cell.

### Post-mortem tissue immunohistochemistry

Sections were dried overnight at 40 °C and immunostaining was performed, following epitope retrieval in citric acid buffer (pH 6) in a pressure cooker for 30 min, using the rabbit anti-MT-ND2 polyclonal antibody (PA5-103952, Invitrogen™ Thermo Fisher Scientific) at a 1:200 dilution and rabbit anti-MT-CO3 antibody (HPA042788, Sigma-Aldrich^®^) at a 1:50 dilution (both incubated for 30 min at room temperature). Immunohistochemical detection was performed using DAB chromogen counterstained with haematoxylin, according to standard operating procedures. The immunohistochemical staining pattern was noted to be dichotomous in nature (*i.e.,* displaying either high intensity or low intensity); consequently, ND2 and CO3 mitochondrial staining intensity was graded semi-quantitatively, as low intensity (‘intensity 1’) or high intensity (‘intensity 2’), in line with other semi-quantitative staining patterns, such as the Allred oestrogen receptor (ER) histoscore used for prognostication in the breast cancer field [69, 101]. The percentage of neurons (*n* = 10 cells per case/control) falling into these staining intensity categories was recorded by neuropathologists, blinded to all demographic and clinical information. Statistical analysis was performed using a two-tailed chi-squared test with Yates’s correction (to reduce approximation error associated with small sample sizes) on integer means of immunoreactivity scores comparing ‘intensity 1′ with ‘intensity 2′ staining profiles across cases and controls (Supplementary Table 3, online resource).

### Data analysis

Statistical analysis was performed using SPSS^®^ Statistics for Windows version 25 (IBM^®^ Corp) or Prism version 8.4.0 (GraphPad Software). Data are presented as mean ± standard error. Data were initially determined to be parametric or non-parametric before applying the appropriate statistical analysis, with false discovery rate (FDR) correction for multiple comparisons, as stated. ^*^p < 0.05, ^**^p < 0.01, ^***^p < 0.001, and ‘ns’ denotes a non-significant result (*p* ≥ 0.05).

### Data availability

The data that support the findings of this study are available from the corresponding authors upon reasonable request.

## Results

### *C9orf72* MNs display dysfunctional axonal homeostasis associated with mitochondrial bioenergetic dysfunction

**Fig. 1:**
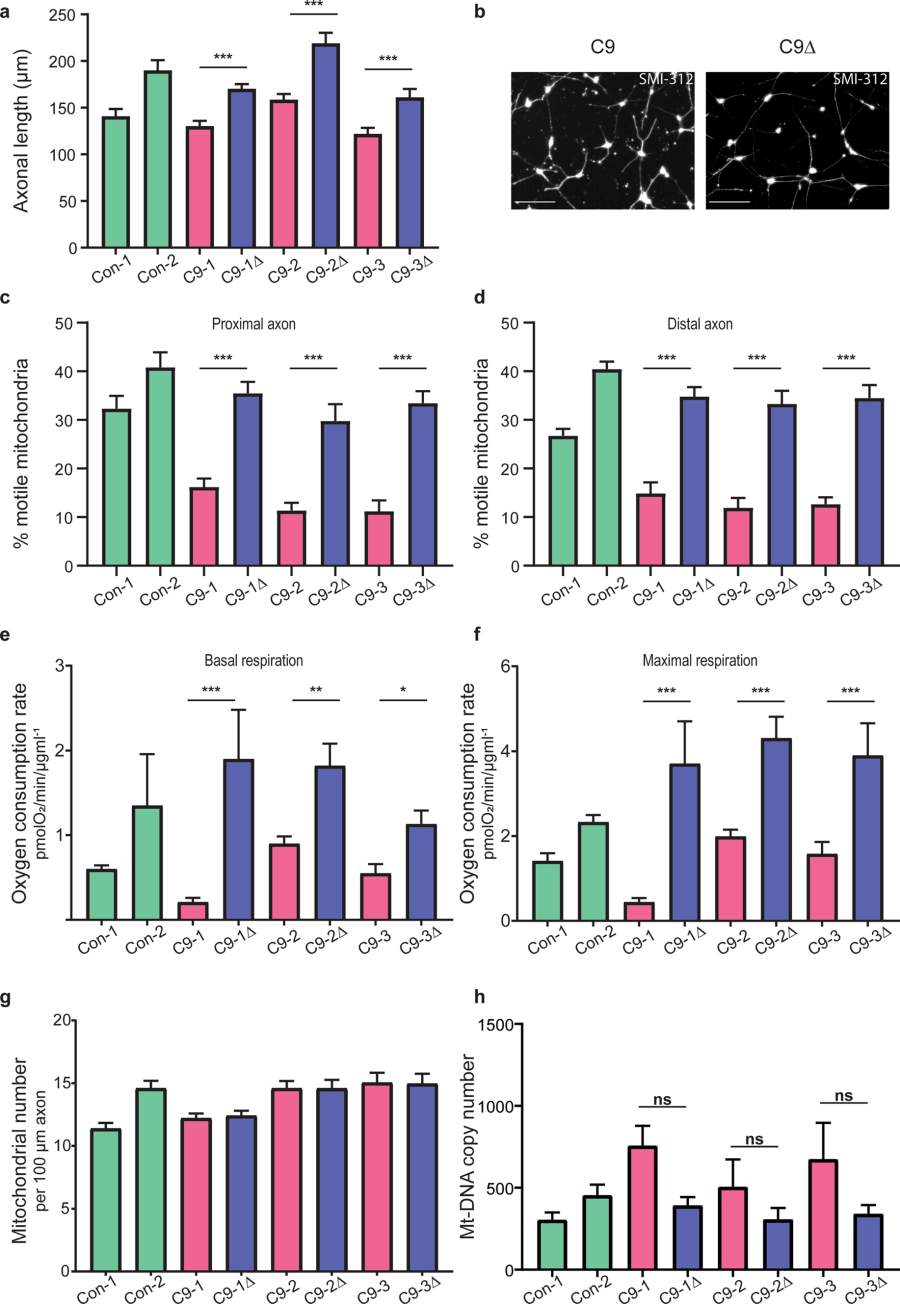
*C9orf72*-dependent dysfunctional axonal homeostasis is associated with impaired mitochondrial bioenergetic function in human iPSC-derived MNs. **a** Quantification of SMI-312 antibody-labelled axonal length in MNs 7 days post platedown (*n* = 50 axons per independent MN differentiation, *N* = 3 independent differentiations; data are represented as mean ± SEM; genotypes used: two independent controls [Con-1, Con-2], three independent patient-derived *C9orf72* lines [C9-1, C9-2, C9-3] with their corresponding isogenic controls [C9-1Δ, C9-2Δ, C9-3Δ]; statistical significance was evaluated using a mixed-effects negative binomial regression with fixed effects for mutant/isogene status and random effects for genotype, with paired mutant-isogene statistics performed via the Mann–Whitney test with FDR correction for multiple comparison testing). **b** Representative images depicting axonal staining via SMI-312 immunocytochemistry of day 7 post platedown *C9orf72* (C9) and paired isogenic control (C9Δ) MNs (scale bars = 100 µm). Quantification of the percentage of motile mitochondria (labelled with mitoDsRed2) relative to the total number of mitochondria in a 100 µm stretch of proximal (**c**) and distal (**d**) axon in *C9orf72-*MNs and isogenic paired controls; two independent healthy controls are shown. Data are represented as mean ± SEM; *n* ≥ 4 axons per independent MN differentiation, *N* = 3 independent differentiations. Statistical significance was evaluated with the Kruskal–Wallis test with FDR correction. Quantification of the oxygen consumption rate (OCR) as measured by the Seahorse Analyzer, normalised to the amount of total protein, denoting basal (**e**) and maximal FCCP-uncoupled (**f**) mitochondrial respiration for *C9orf72-*MNs and isogenic paired controls; two independent healthy controls are also shown. Data are represented as mean ± SEM; *n* ≥ 4 wells per line per experiment, with experiments repeated in three independent cultures from different differentiations (*N* = 3). Statistical significance was evaluated with one-way ANOVA with post hoc mutant-isogene paired FDR-corrected *t* tests. **g**, Quantification of the axonal mitochondrial number in a 100 µm stretch of distal axon in *C9orf72-*MNs and isogenic paired controls; two independent healthy controls are also shown. Data are represented as mean ± SEM; *n* = 10 axons per line per experiment, with experiments repeated in three independent cultures from different differentiations (*N* = 3). Statistical significance was evaluated with one-way ANOVA. **h** Quantification of mitochondrial DNA copy number in *C9orf72-*MNs and isogenic paired controls; two independent healthy controls are also shown. Data are represented as mean ± SEM, with experiments repeated in at least three independent cultures from different differentiations (*N* ≥ 3). Statistical significance was evaluated with the Kruskal–Wallis test with FDR correction. *p < 0.05, **p < 0.01, ***p < 0.001, ‘ns’ denotes non-significant result (*p* ≥ 0.05)

We generated highly enriched spinal cord MNs from three independent *C9orf72* iPSC lines and their corresponding isogenic controls, as previously reported by our group [92]. To investigate the consequence of the *C9orf72* repeat expansion mutation on MN axonal homeostasis, we first assessed axonal morphology and transport. We observed that *C9orf72* MNs, when compared with their isogenic controls, have significantly shorter axons (Fig. 1a, 1b; Supplementary Fig. 1, online resource), and fewer motile mitochondria in both the proximal (Fig. 1c) and distal (Fig. 1d) axon, as assessed by live imaging of MNs transduced with mitoDsRed2 (Supplementary Video 1a, 1b, online resource). To investigate for the presence of associated deficits in MN cellular metabolism, we profiled *C9orf72* MNs cellular energetics (Supplementary Fig. 2a, 2b, online resource). We found impaired basal (Fig. 1e) and maximal (Fig. 1f) mitochondrial respiration, despite normal axonal mitochondrial number (Fig. 1g) and mitochondrial copy number (Fig. 1h), implicating abnormalities in the electron transport chain machinery and/or mitochondrial substrate provision, necessary for maintenance of electron flow. Undifferentiated iPSCs did not demonstrate the mitochondrial deficit (Supplementary Fig. 2c, 2d, online resource), implying neuronal specificity. We also observed no change in the glycolytic function in the *C9orf72* MNs, suggesting that glycolysis is neither involved in a negative nor compensatory fashion (Supplementary Fig. 2e, 2f, online resource).

### Transcriptomic analysis of *C9orf72* MNs reveals reduced expression of mitochondrially encoded transcripts of the mitochondrial respiratory chain

**Fig. 2:**
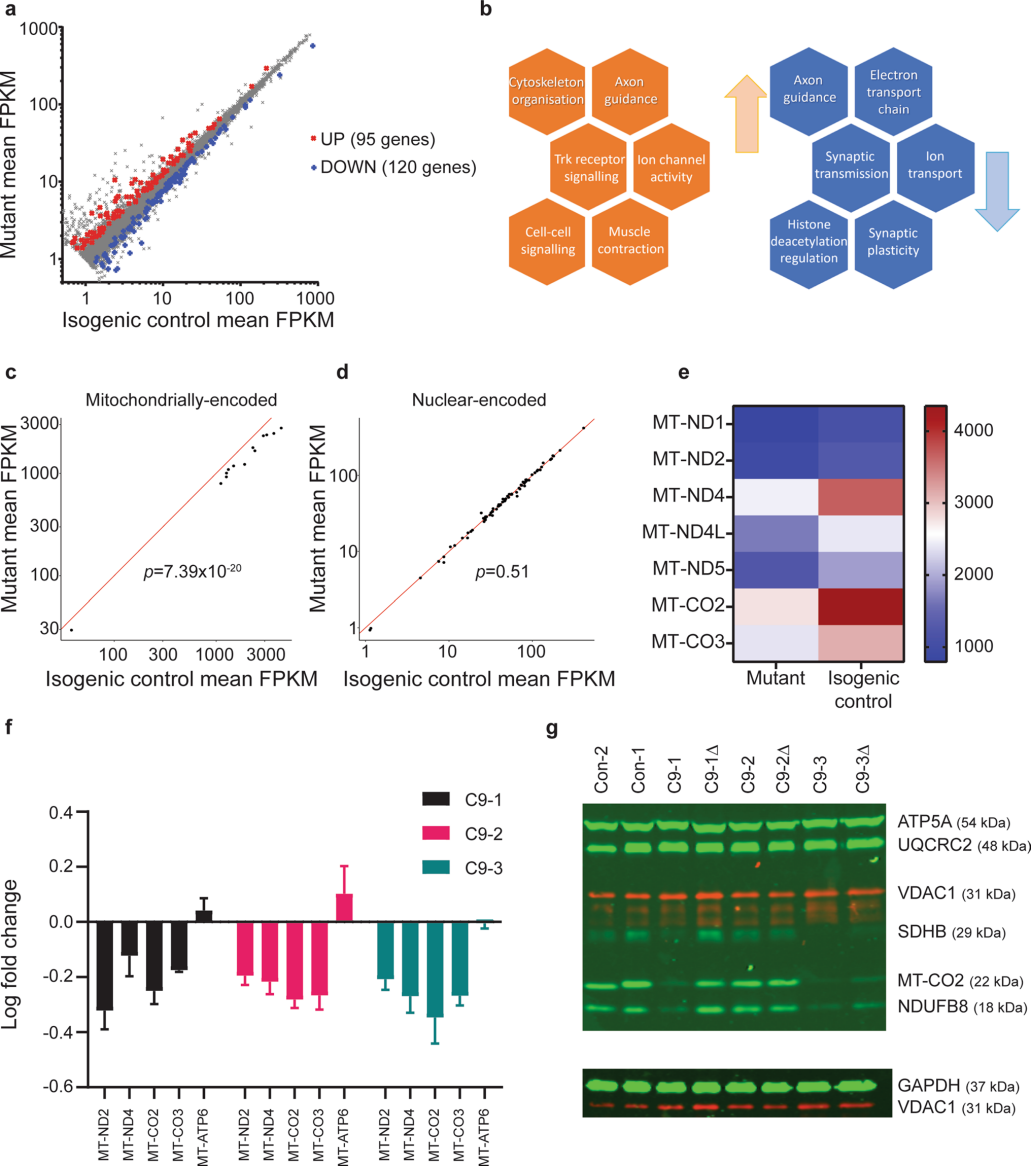
Transcriptomic analysis revealed reduced gene expression of the mitochondrial electron transport chain in human iPSC-derived *C9orf72*-MNs. **a** Results of differential gene expression analysis, using DESeq2, examining the intersection in commonly and concordantly differentially expressed genes between the two mutant-isogene pairs, using an FDR of 20%, and discarding genes with an average FPKM < 1. The scatter plot shows the comparison of gene expression (as average FPKM). Red and blue data points denote the overlap of significantly up- and down-regulated genes in both mutant-correction pairs, respectively. **b** Summary of notable results from gene ontology analysis performed on the differentially expressed genes that identified putatively up (red) and down (blue) regulated altered pathways or processes. Average FPKM scatter plots comparing gene expression in *C9orf72* mutant *versus* isogenic controls for two gene sets of interest (**c**, mitochondrially encoded mitochondrial transcripts and (**d**) nuclear-encoded mitochondrial transcripts). Competitive gene set testing using CAMERA, examining the differential gene expression for these two gene sets of interest compared with all other genes, showed that mitochondrially encoded transcripts were down-regulated (*p* = 7.39 × 10^–20^), whereas nuclear-encoded mitochondrial transcripts were not (*p* = 0.51). **e** Heatmap summarising the average gene expression (FPKM) of significantly dysregulated genes of complex I (MT-ND1, MT-ND2, MT-ND4, MT-ND4L, MT-ND5) and complex IV (MT-CO2, MT-CO3) subunits of the electron transport chain for *C9orf72 versus* isogenic control MNs. **f** Log_10_ mean fold change ± SEM of gene expression normalised to the respective isogenic control determined via RT-PCR for C9-1 pair (black bars), C9-2 pair (red bars), and C9-3 pair (green bars) for MT-ND2 and MT-ND4 subunits of complex I; MT-CO2 and MT-CO3 subunits of complex IV; and MT-ATP6 subunit of complex V. **g** Representative image of western blot of protein lysates from two independent controls [Con-1, Con-2], three independent patient-derived *C9orf72* lines [C9-1, C9-2, C9-3] with corresponding isogenic controls [C9-1Δ, C9-2Δ, C9-3Δ] using a primary Total OXPHOS cocktail antibody against multiple mitochondrial electron transport chain subunits. Bands depict protein expression of complex I (NDUFB8; reduced), complex II (SDHB), complex III (UQCRC2), complex IV (MT-CO2; reduced) and complex V (ATP5A), and housekeeper proteins against GAPDH and mitochondrial outer membrane protein, VDAC-1

To identify putative dysregulated molecular pathways that may contribute to these axonal morphological and functional deficits, we next examined the *C9orf72* MN transcriptome by performing RNA-seq on two pairs of *C9orf72* and isogenic control MNs, and two independent controls. We observed a high degree of similarity between the transcriptome of mutant *C9orf72* MNs and their corresponding isogenic control (Supplementary Fig. 3a, online resource). Differential gene expression analysis identified 215 dysregulated transcripts (95 upregulated and 120 downregulated) (Fig. 2a; Supplementary Data 1, online resource). Gene ontology analysis revealed, *inter alia*, dysregulation in pathways implicated in axonal homeostasis and oxidative phosphorylation (Fig. 2b; Supplementary Data 1, online resource).

It is known that mitochondrial proteins are nuclear-encoded and mitochondrial DNA-encoded. Intriguingly, we found the mitochondrially encoded transcripts to be specifically downregulated in *C9orf72* MNs using competitive gene set testing, where two gene sets of interest (for mitochondrial DNA-encoded mitochondrial proteins and nuclear-encoded mitochondrial proteins) were each compared with all other genes; mitochondrially encoded transcripts (*p* = 7.39 × 10^–20^) but not nuclear-encoded mitochondrial transcripts (*p* = 0.51) were significantly down-regulated (Fig. 2c, d). Furthermore, the nine mitochondrially encoded transcripts (contributing to complexes I and IV of the electron transport chain, and the mitochondrial small and large ribosomal subunits; Fig. 2e) whose expression was reduced are all present on the heavy strand of the mitochondrial DNA duplex, whereas the light strand transcripts, which notably encode for mitochondrial tRNAs, were unaltered (Supplementary Fig. 3b, online resource).

Quantitative RT-PCR of a representative panel of predicted dysregulated (MT-ND2 and MT-ND4 subunits of electron transport chain complex I, and MT-CO2 and MT-CO3 subunits of complex IV) and unaltered (MT-ATP6 subunit of complex V) mitochondrially encoded transcripts, and western blotting analysis of five OXPHOS complexes, were next undertaken from all three mutant-isogene *C9orf72* MN pairs and two independent controls. This confirmed dysregulation of various subunits of the electron transport chain, most notably complex I and IV (Fig. 2f, g; Supplementary Fig. 3c, online resource).

We next performed promoter analysis on the dysregulated genes to identify putative common transcriptional factor family regulatory control (Supplementary Data 1, online resource). This showed an overrepresentation of potential transcription factor binding site motifs for the nuclear respiratory factor 1 (NRF1) transcription factor family, when compared with all other transcription factor families (Promoter *Z* score = 31.87).

### Examination of human *C9orf72* post-mortem spinal cord tissue using BaseScope™ shows MN-selective reduced expression of complexes I and IV of the mitochondrial electron transport chain

**Fig. 3:**
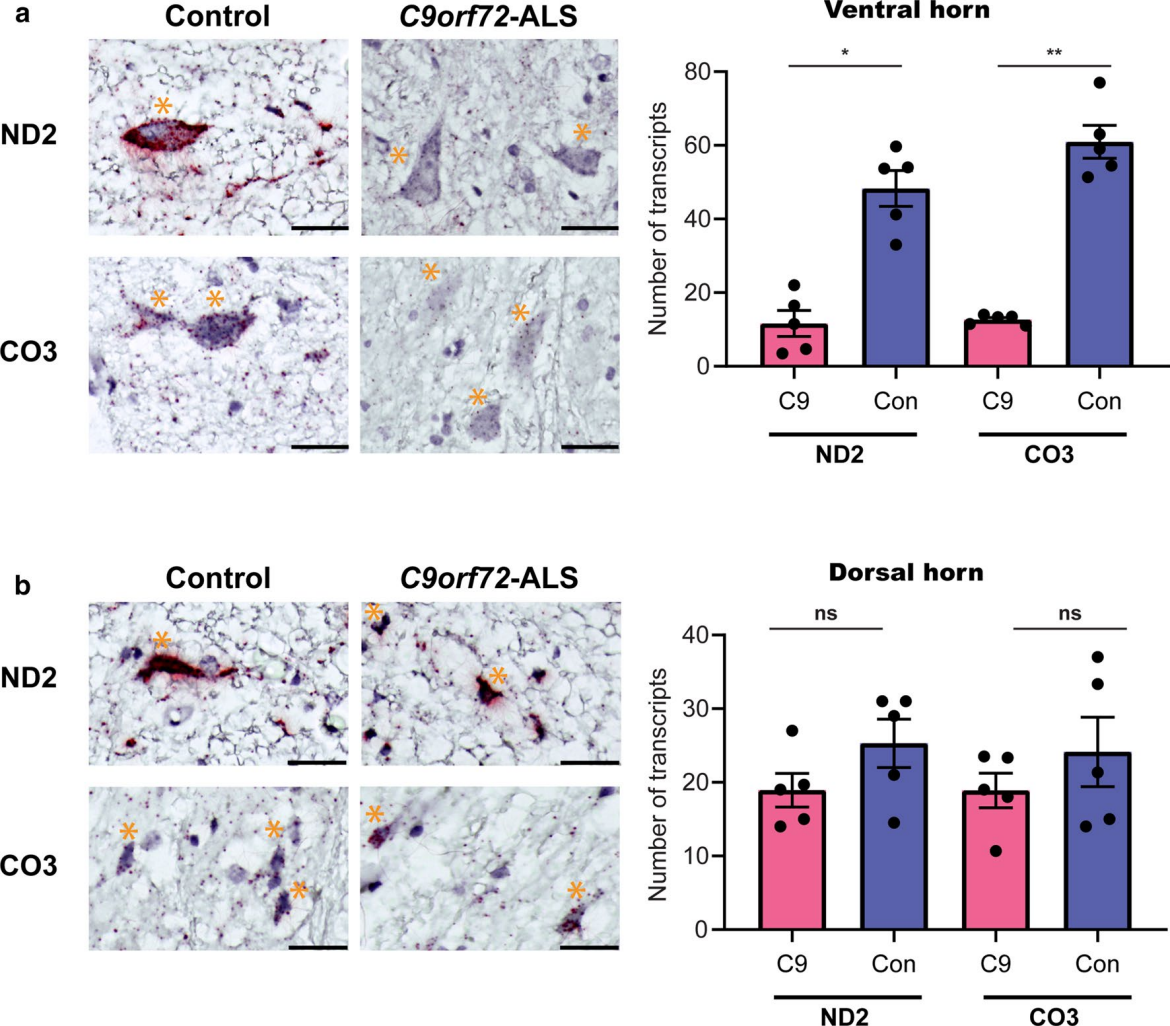
Examination of human *C9orf72* post-mortem spinal cord tissue shows reduced expression of complexes I and IV of the mitochondrial electron transport chain in ventral horn motor, but not dorsal horn sensory, neurons. **a** Representative photomicrographs showing reduced expression of MT-ND2 (complex I) and MT-CO3 (complex IV) transcripts in ventral horn motor neurons of the post-mortem spinal cord from a *C9orf72* hexanucleotide repeat expansion mutation case compared with its age- and sex-matched control. Red spots highlight individual mRNA molecules of MT-ND2 or MT-CO3. Tissue was counterstained with haematoxylin. Scale bars = 50 µm. Orange asterisk denotes an anterior horn motor neuron. Bar chart depicts the quantification of the number of MT-ND2 and MT-CO3 transcripts per ventral horn spinal motor neuron. Bars represent aggregate mean ± SEM, with each dot representing the mean count for between one and five cells for each of the five post-mortem specimens for *C9orf72*-ALS (red bars) and their age- and sex-matched controls (blue bars). Statistical significance was evaluated with the Kruskal–Wallis test with FDR correction. **b** Representative photomicrographs of the dorsal horn sensory neurons, examined using the same probes that recognise individual mRNA of MT-ND2 or MT-CO3, showing comparable expression between the same cases and controls. Scale bars = 50 µm. Orange asterisk denotes a dorsal horn sensory neuron. Bar chart depicts the quantification of the number of MT-ND2 and MT-CO3 transcripts per dorsal horn neuron. Bars represent aggregate mean ± SEM, with each dot representing the mean count for between one and five cells for each of the five post-mortem specimens for *C9orf72*-ALS (red bars) and their age- and sex-matched controls (blue bars). Statistical significance was evaluated with the Kruskal–Wallis test with FDR correction for multiple comparisons. *p < 0.05, **p < 0.01, ‘ns’ denotes non-significant result (*p* ≥ 0.05)

To evaluate the in vivo relevance and selectivity of the observed in vitro transcriptional signature, we performed neuropathological analysis of human spinal cord motor neurons and sensory neurons. We used BaseScope™ to examine the expression of complex I (MT-ND2) and IV (MT-CO3) transcripts, in post-mortem samples from *C9orf72*-ALS cases (*N* = 5) and age- and sex-matched non-neurological controls (*N* = 5). We found significantly reduced expression of both MT-ND2 and MT-CO3 in ventral horn spinal motor neurons (Fig. 3a; Supplementary Fig. 4, online resource), but not in dorsal horn sensory neurons (Fig. 3b; Supplementary Fig. 4, online resource). Thus, these data confirm, in human autopsy cases, that the *C9orf72* repeat expansion mutation results in down-regulation of genes associated with the mitochondrial electron transport chain, and that this dysregulation is selective to anterior horn (motor) neurons. Consistent with this transcriptional signature, we observed concomitant changes at the level of protein expression (Supplementary Fig. 5, online resource).

**Fig. 4:**
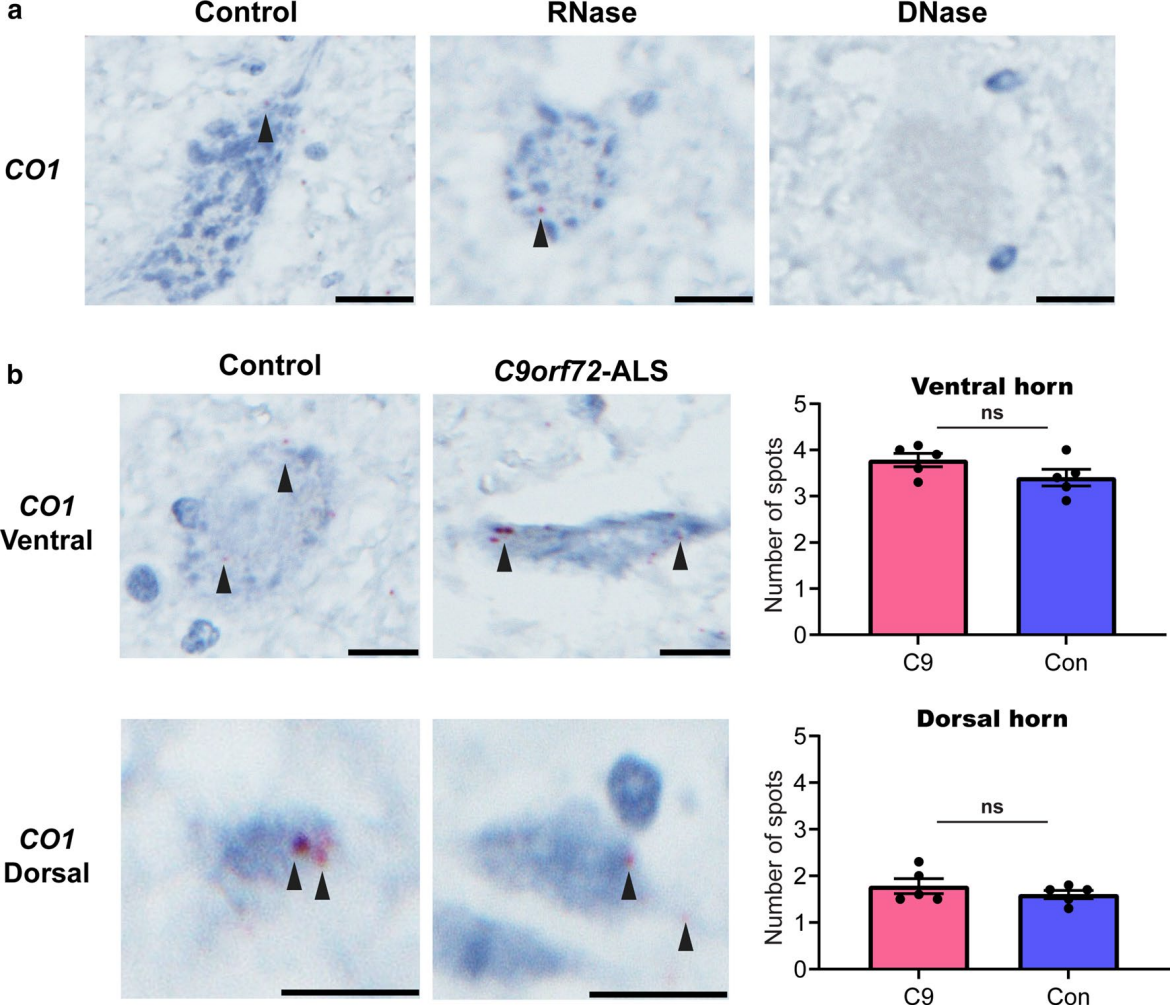
Mitochondrial DNA copy number in human *C9orf72* post-mortem spinal cord tissue is unaltered. **a** Representative photomicrographs showing DNA in situ hybridisation in control ventral horn spinal cord tissue. Red spots denote probe binding to *MT-CO1*—a surrogate of mitochondrial DNA copy number. RNase treatment resulted in no change in signal, while DNase treatment resulted in elimination of signal, confirming that the probe binds to DNA, and not RNA. Tissue was counterstained with haematoxylin and images were acquired at 40 × magnification. Scale bars = 20 µm. **b** Representative photomicrographs showing unaltered mitochondrial copy number in a *C9orf72* hexanucleotide repeat expansion mutation case compared with its age- and sex-matched control, both in spinal cord ventral horn motor neurons (upper panel) and dorsal horn sensory neurons (lower panel). Red spots denote probe binding to *MT-CO1*. Tissue was counterstained with haematoxylin and images were acquired at 40 × magnification. Scale bars = 20 µm. Bar charts depict the quantification of the number of spots per neuron in either the ventral (upper panel) or dorsal horn (lower panel). Bars represent aggregate mean ± SEM, with each dot representing the mean count for ten cells for each of the five post-mortem specimens for *C9orf72*-ALS (red bars) and their age- and sex-matched controls (blue bars). Statistical significance was evaluated with the Mann–Whitney test; ‘ns’ denotes non-significant result (*p* ≥ 0.05)

To exclude the possibility of reduced mitochondrial DNA driving the transcriptional signature in *C9orf72*-ALS MNs, we next quantified mitochondrial DNA copy number through a BaseScope™ DNA in situ hybridisation technique that uses a probe targeting the antisense strand of the *MT-CO1* gene (see Materials and Methods). This showed unaltered mitochondrial copy number in neurons from cases *versus* controls, both in the ventral and dorsal horn spinal cord (Fig. 4).

### *C9orf72*-dependent dysfunctional axonal homeostasis is caused by mitochondrial bioenergetic dysfunction

Peroxisome proliferator-activated receptor gamma coactivator 1α (PGC1α) is a master regulator of mitochondrial energy metabolism and biogenesis [57], which are usually inextricably coupled [111]. PGC1α overexpression, through the modulation of the activity of multiple transcription factors (including NRF1), increases the expression of mitochondrial DNA-encoded and nuclear-encoded mitochondrial proteins [111], and it improves individual mitochondrial function [6], which, in turn, stimulates mitochondrial biogenesis [7, 88, 102]. We therefore hypothesised that overexpressing PGC1α, thereby increasing cellular bioenergetic function and mitochondrial biogenesis, may be sufficient to rescue the observed axonal phenotypes in *C9orf72* MNs.

**Fig. 5:**
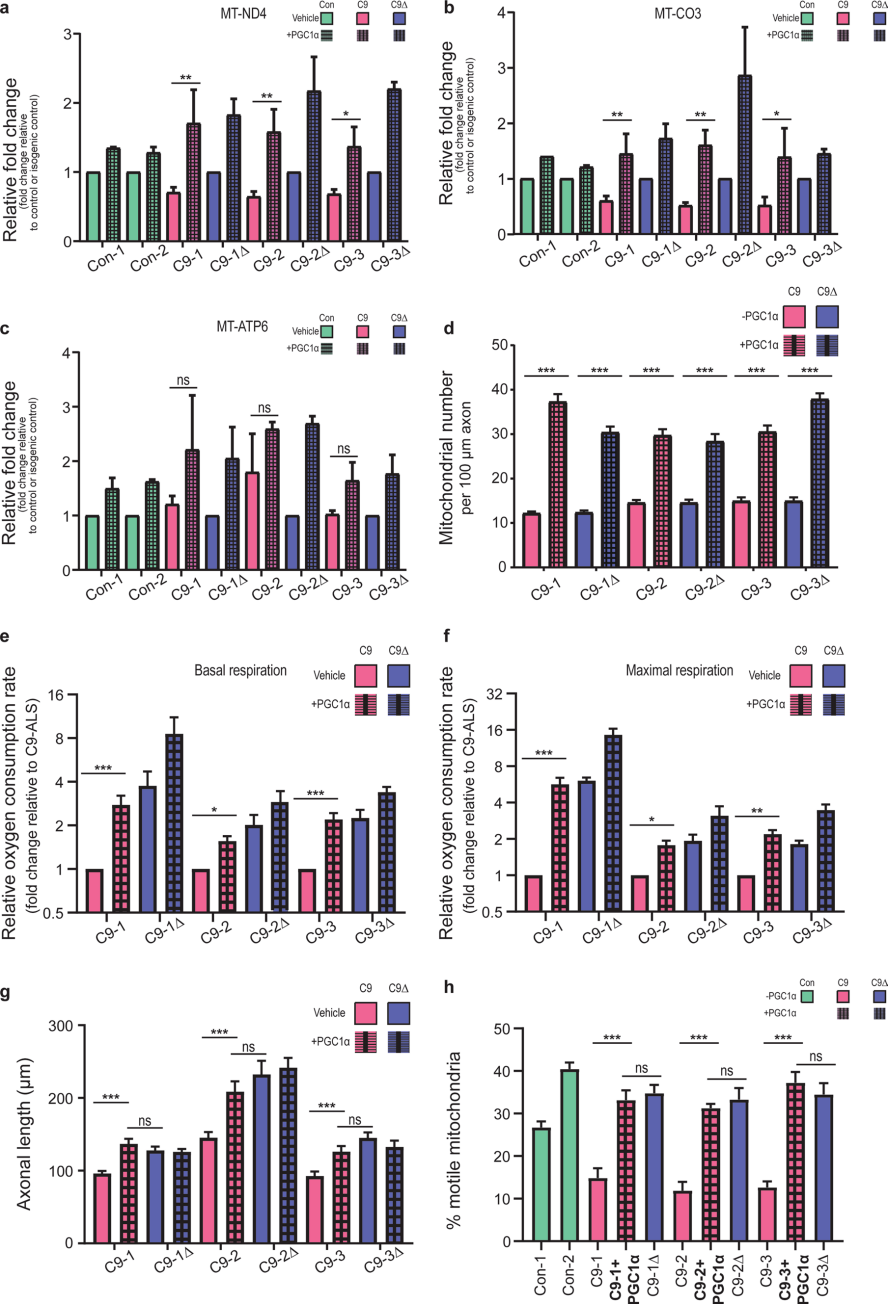
*C9orf72*-dependent dysfunctional axonal homeostasis is caused by mitochondrial bioenergetic dysfunction in human iPSC-derived MNs. **a**–**c** Mean fold change ± SEM of gene expression normalised to the respective isogenic control (or control) determined via RT-PCR; genotypes used: two independent controls [Con-1, Con-2], three independent patient-derived *C9orf72* lines [C9-1, C9-2, C9-3; red bars] with their corresponding isogenic controls [C9-1Δ, C9-2Δ, C9-3Δ; blue bars], with (hatched bars) and without (unhatched bars) PGC1α overexpression, for transcripts: MT-ND4 (complex I; **a)**, MT-CO3 (complex IV; **b)**, and MT-ATP6 (complex V; **c**). **d** Quantification of the axonal mitochondrial number in a 100 µm stretch of distal axon in *C9orf72-*MNs and their respective isogenic controls, with (hatched bars) and without (unhatched bars) PGC1α overexpression. Data are represented as mean ± SEM; *n* ≥ 4 axons per line per experiment, with experiments repeated in three independent cultures from different differentiations (*N* = 3). Statistical significance was evaluated with one-way ANOVA with post hoc mutant-isogene paired FDR-corrected *t* tests. Quantification of the relative oxygen consumption rate (OCR) as measured by the Seahorse Analyzer, normalised to the amount of total protein, denoting basal (**e**) and maximal FCCP-uncoupled (**f**) mitochondrial respiration for *C9orf72-*MNs and isogenic paired controls, with (hatched bars) and without (unhatched bars) PGC1α overexpression. Data are represented as mean fold change (relative to *C9orf72*-ALS) ± SEM; *n* ≥ 4 wells per line per experiment, with experiments repeated in three independent cultures from different differentiations (*N* = 3). Statistical significance was evaluated with one-way ANOVA with post hoc mutant-isogene paired FDR-corrected *t* tests. **g** Quantification of the SMI-312 antibody-labelled axonal length in MNs 7 days post platedown, with (hatched bars) and without (unhatched bars) PGC1α overexpression (*n* > 15 axons per independent MN differentiation, *N* = 3 independent differentiations; data are represented as mean ± SEM; genotypes used: three independent patient-derived *C9orf72* lines [C9-1, C9-2, C9-3] with their corresponding isogenic controls [C9-1Δ, C9-2Δ, C9-3Δ]; statistical significance was evaluated using the Kruskal–Wallis test with FDR correction). **h** Quantification of the percentage of motile mitochondria (labelled with mitoDsRed2) relative to the total number of mitochondria in a 100 µm stretch of axon in *C9orf72-*MNs, with (hatched bars) and without (unhatched bars) PGC1α overexpression, and isogenic paired controls; two independent healthy controls are also shown. Data are represented as mean ± S.E.M; *n* ≥ 4 axons per independent MN differentiation, *N* = 3 independent differentiations. Statistical significance was evaluated with the Kruskal–Wallis test with FDR correction. *p < 0.05, **p < 0.01, ***p < 0.001, ‘ns’ denotes non-significant result (*p* ≥ 0.05)

Accordingly, we performed lentiviral-mediated overexpression of PGC1α, the expression of which was itself unchanged basally between mutants and isogenic *C9orf72* MNs (Supplementary Fig. 6, online resource). This resulted in the restoration of the expression of mitochondrial transcripts (complex I: MT-ND4 and complex IV: MT-CO3) of the electron transport chain (Fig. 5a–c), together with an increase in mitochondrial number in the distal axon (Fig. 5d). This in turn led to rescue of the *C9orf72*-dependent impairment in basal (Fig. 5e) and maximal (Fig. 5f) mitochondrial function. Critically, boosting mitochondrial bioenergetics was sufficient to rescue both the axonal length (Fig. 5g) and mitochondrial transport (Fig. 5h; Supplementary Video 2a, 2b, online resource) phenotypes observed in *C9orf72* MNs. Taken together, these data establish a novel mechanistic link between mitochondrial bioenergetic dysfunction and axonal dysfunction in the pathophysiology of *C9orf72*-ALS.

## Discussion

Our human in vitro and neuropathological post-mortem findings establish, for the first time, the presence of metabolic deficits in *C9orf72*-ALS MNs, owing to loss of mitochondrial bioenergetic function. Through the use of isogenic controls, and pathway manipulation derived from unbiased transcriptomics, we further show a novel causal and mechanistic link between MN bioenergetic failure and axonal dysfunction. Given the particular vulnerability of MNs to metabolic stress [52], our findings suggest a cell-autonomous mechanism to explain the selective MN degeneration found in ALS [74], and further confirm that ameliorating mitochondrial function is an area of therapeutic promise [66].

It is well established from animal models of ALS that targeting the axon leads to delayed onset of disease and improved survival [90, 93, 95] and that preventing MN loss alone, but not axonal degeneration, is inadequate for promoting survival [82]. Given that importance of maintenance of axonal homeostasis, it is thus important to better understand the impact of the *C9orf72* repeat expansion mutation on human MN axonal properties. In the present study, we found two axonal phenotypes underlying dysfunctional axonal homeostasis in *C9orf72* MNs. First, we demonstrate a novel phenotype of impaired fast axonal transport of mitochondrial cargo in *C9orf72* MNs. Second, we discovered a novel morphological deficit—of shorter MN axonal length—consistent with phenotypic observations across other, rarer, ALS mutations, such as *SOD-1* [20, 48] and *TDP-43* [17, 33, 34, 49], as well as sporadic ALS [36]. While to our knowledge this is the first report of these phenotypes in *C9orf72* human iPSC-derived MNs, reduced mitochondrial trafficking has recently been shown in primary cortical excitatory neurons in an inducible mouse model of poly(GR) toxicity [22]. The precise underlying pathological mechanisms of *C9orf72*-ALS remain unclear, with non-mutually exclusive C9orf72 haploinsufficiency and toxic gain of function mechanisms implicated. Recent work from Sivadasan et al*.* showed modulation of C9orf72 protein levels in mouse primary MNs influences actin cytoskeletal dynamics and axon outgrowth [96]. However, in neurons, the primary mediator of long-range mitochondrial transport is via microtubule-based motor machinery. In addition, it has been recently showed that C9orf72 loss of function alone does not contribute to lysosomal fast axonal transport deficits in *C9orf72* iPSC-derived MNs [1]. Previous *C9orf72* human iPSC studies by ourselves [92, 115], and others [1], showing pathological dipeptide repeat proteins and RNA foci, but not reduction in expression of *C9orf72* (both at transcriptomic and protein levels), suggest that the observed dysfunctional axonal homeostasis is due to toxic gain of function mechanisms [16].

A key component of either the dying backward [35, 71] or the dying forward model [15] of neurodegeneration is axonal transport [8]. Thus, perturbations in axonal transport are key mediators of neurodegeneration, warranting an improved understanding of upstream mechanisms. Mitochondria are transported via fast axonal transport to the distal neurites and provide the necessary energy for local axonal processes that shape neuronal wiring and axon maintenance. Microtubules are critical, as are two main motor proteins—kinesin (for anterograde transport) and dynein (for retrograde transport)—in conjunction with axonal actin filaments [45]. The processes involved are all highly energy consuming, raising the question of whether impairments in energy metabolism contribute to defective axonal transport. While impairments in microtubule machinery have been observed in a rare ALS-causing mutation, *FUS,* leading to axonal transport deficits [41], metabolic impairments were unexpectedly shown to be absent [104]. Indeed, until the present study, it has not been shown that ALS patient-derived MNs metabolically differ from healthy MNs. In contrast, we report a cell-autonomous bioenergetic functional deficit in ALS human iPSC-derived MNs. Mitochondrial metabolic function was impaired, with neither a concomitant difference in glycolysis, nor altered metabolic state in the parental iPSC lines. Unbiased bulk RNA-sequencing and transcriptomic analysis revealed a distinct mitochondrial signature, with reduction in expression of mitochondrial-DNA encoded transcripts relating to the electron transport chain. Importantly, this mitochondrial loss of function signature was recapitulated in patient spinal cord tissue in a selective manner; thus, ventral horn motor neurons—but not dorsal horn sensory neurons—displayed down-regulation of expression of transcripts relating to complexes I and IV of the electron transport chain. In addition to the observation of no difference in expression in the dorsal horn neurons (acting as an internal control), previous findings of an upregulation of the *GRIA1* transcript in ventral spinal MNs from the same *C9orf72*-ALS cases [40, 92] suggest that the observed down-regulation of mitochondrial gene expression in ventral horn spinal motor neurons is not due to a non-specific post-mortem artifact. Furthermore, mitochondrial copy number is known to be unchanged in ALS cases in general compared to controls [108]. Indeed, our in vitro and post-mortem findings together also exclude the possibility of a reduction in mitochondrial copy number in *C9orf72*-ALS. Consistent with these findings, we did not observe significant changes in either axonal mitochondrial counts or transcript levels of mitochondrial tRNAs in *C9orf72* MNs. The trend towards increased mitochondrial copy number observed in *C9orf72*-MNs may reflect a compensatory response to the mitochondrial transcriptional dysregulation. Taken together, we conclude that the observed reduced mitochondrial gene expression signature and concomitant metabolic deficit are not mechanistically driven by reduced mitochondrial DNA.

The molecular basis of how the *C9orf72* repeat expansion mutation results in transcriptional dysregulation of mitochondrial electron transport chain genes is unclear. Future studies will also need to evaluate mitochondrial transcriptional status in non-*C9orf72* cases, including sporadic ALS, as well as in other forms of motor neuron disease, such as spinal muscular atrophy [14], to begin to address whether our findings are directly related to *C9orf72* pathogenic mechanisms, such as dipeptide repeat proteins and/or due to, for example, TDP-43 aggregation. Indeed, recent findings show that *C9orf72* dipeptide repeat proteins can lead to cytoplasmic TDP-43 aggregation [24, 99]. Thus, does TDP-43 pathology, observed in the vast majority of ALS cases, including *C9orf72* [4, 72], modulate mitochondrial homeostasis by regulating the processing of mitochondrial transcripts [46]? Indirect evidence for this idea is supported by recent findings of transcriptional dysregulation in pathways involved in mitochondrial function and energy production in TDP-43-depleted motor neurons [17]. Moreover, TDP-43 has been shown to exert toxicity by entering mitochondria and specifically impairing OXPHOS complex I via the preferential binding of mitochondrially transcribed ND3/6 mRNAs and by inhibiting their translation to cause mitochondrial dysfunction and neuronal loss [107], as well as triggering mitochondrial DNA release and neuroinflammatory cGAS/STING activation [112]. Finally, recent work by Onesto et al*.* showed that there is compensatory mitochondrial biogenesis, as evidenced by PGC1α upregulation, in dermal fibroblasts derived from patients with *C9orf72*-ALS [77]. This suggests that, despite carrying the pathological mutation, most cell types can compensate for the mitochondrial dysfunction by stimulating biogenesis, and selective vulnerability could arise partly from an inability of motor neurons to modulate this homeostatic mechanism.

Crucially, notwithstanding an unaltered NRF1-PGC1α axis basally in *C9orf72* MNs, we were able to boost mitochondrial function (and biogenesis) through manipulation of its master regulator, PGC1α, leading to rescue of the axonal phenotypes, providing a novel mechanistic link between mitochondrial bioenergetics and axonal dysfunction. Fast axonal transport is a highly energy demanding process [97]. Chronic dysfunction of the mitochondrial electron transport chain could result in a reduction of global ATP levels in MNs. Consequently, motor proteins would arrest, given the cellular milieu being deprived of adequate ATP. The successful manipulation of a key pathway involved in mitochondrial energy metabolism adds to the emerging spinal cord injury literature that has shown that restoring cellular energetics and axonal transport promotes axonal outgrowth and regeneration [42]. Our data suggest that targeting this tractable pathway may thus also be highly relevant to neurodegeneration [56, 75], accepting the possibility of there being other competing mechanisms driving the dysfunctional axonal homeostasis observed in *C9orf72* MNs.

Our findings add important new metabolic functional data to the existing ALS mitochondrial literature. Early, elegant post-mortem studies (well before the current rich genetic landscape of ALS had been determined) showed that patients with ALS have dense clusters of mitochondria in the anterior horn of the lumbar spinal cord [87] and presynaptic mitochondrial swelling in their motor neurons [94]. The cellular distribution of mitochondria was also shown to be affected, with the majority of mitochondria being located in the soma and proximal axon [86]. Moreover, the total amount of mitochondrial DNA, measured by Southern blot, was reduced in the spinal cord from sporadic ALS patients [109], associated with a decrease in the activity of the electron transport chain complexes in spinal cord mitochondria [109] and a reduction in the activity of key mitochondrial enzymes [13]. Our study provides a mechanistic link between the functional MN metabolic deficits to alterations in gene expression. More recently, astrocytic metabolic inflexibility [3] and mitochondrial aberrations in patient-derived fibroblasts [77] and iPSC models [27, 36, 59] have been demonstrated in *C9orf72-*ALS. *C9orf72* patient-derived motor neurons have swollen mitochondria on electron microscopy [27], a reduced [27] or increased [59] mitochondrial membrane potential, associated with altered calcium buffering, contributing to glutamate excitotoxity [26], and elevated levels of cytochrome c [27]. They also show increased age-dependent oxidative stress associated with poly(GR) dipeptide repeat proteins binding to mitochondrial ribosomal proteins [59], and more recently in *Drosophila* muscle, poly(GR) has been shown to interact with components of the ‘mitochondrial contact site and cristae organising system’ impacting on mitochondrial ion homeostasis [54]. In our study, we show a distinct mitochondrial bioenergetic deficit in *C9orf72* MNs. In addition to calcium buffering, mitochondrial bioenergetics could also contribute to neuronal vulnerability to excitotoxicity. PGC1α, which controls cellular energetics including mitochondrial biogenesis, has been shown to negatively regulate glutamate receptor mediated excitotoxicity in Huntington’s disease mouse models [79]. Indeed, we have previously shown that *C9orf72* MNs display vulnerability to excitotoxicity, owing to dysregulation in glutamate receptors [92], and the present findings raise the possibility of there being positive feedback on the excitotoxic signalling pathways. Finally, a large screening study (> 1200 drugs) from 32 sporadic ALS human iPSC lines showed that ropinirole, a dopamine (D2) receptor agonist used to treat Parkinson’s disease motor symptoms, reverses their identified neurite length, cytotoxicity and abnormal protein aggregations phenotypes, by reducing the elevated reactive oxygen species, implicating ropinirole’s non-canonical effects on mitochondrial pathways [36].

In summary, our study establishes a novel mechanistic link between mitochondrial dysfunction and axonal dysfunction in ALS using human-based disease modelling. It is conceivable that boosting mitochondrial metabolism locally at the growth cone or synapses could improve the size of the motor unit by promoting terminal axonal sprouting and/or increasing stability of the neuromuscular junction. Accordingly, we believe that our findings highlight the importance of targeting mitochondrial dysfunction directly, to discover novel therapeutic agents for this devastating disease [66].

## Electronic supplementary material

Below is the link to the electronic supplementary material.

Supplementary Figures and Tables (PDF 2645 kb)

Supplementary Data 1 (XLSX 171 kb)

Supplementary Video 1a (AVI 1169 kb)

Supplementary Video 1b (AVI 3051 kb)

Supplementary Video 2a (AVI 323810 kb)

Supplementary Video 2b (AVI 216879 kb)
