# Supplementary material for: Mitochondrial bioenergetic deficits in C9orf72 amyotrophic lateral sclerosis motor neurons cause dysfunctional axonal homeostasis
Source: Acta Neuropathol. 2021 Jan 4;141(2):257–79. doi: 10.1007/s00401-020-02252-5 (PMC7847443; doi:10.1007/s00401-020-02252-5)
Supplement: Supplementary file 1 — Supplementary Figures and Tables (PDF 2645 kb) [file 401_2020_2252_MOESM1_ESM.pdf]

SUPPLEMENTARY DATA & LEGENDS

Mitochondrial bioenergetic deficits in *C9orf72* amyotrophic lateral sclerosis motor neurons cause dysfunctional axonal homeostasis

Arpan R. Mehta, Jenna M. Gregory, Owen Dando, Roderick N. Carter, Karen Burr, Jyoti Nanda, David Story, Karina McDade, Colin Smith, Nicholas M. Morton, Don J. Mahad, Giles E. Hardingham, Siddharthan Chandran* & Bhuvaneish T. Selvaraj*

Supplementary Figures 1-6

Supplementary Tables 1-3

Supplementary Data Table

Supplementary Videos 1-2

Supplementary Fig 1

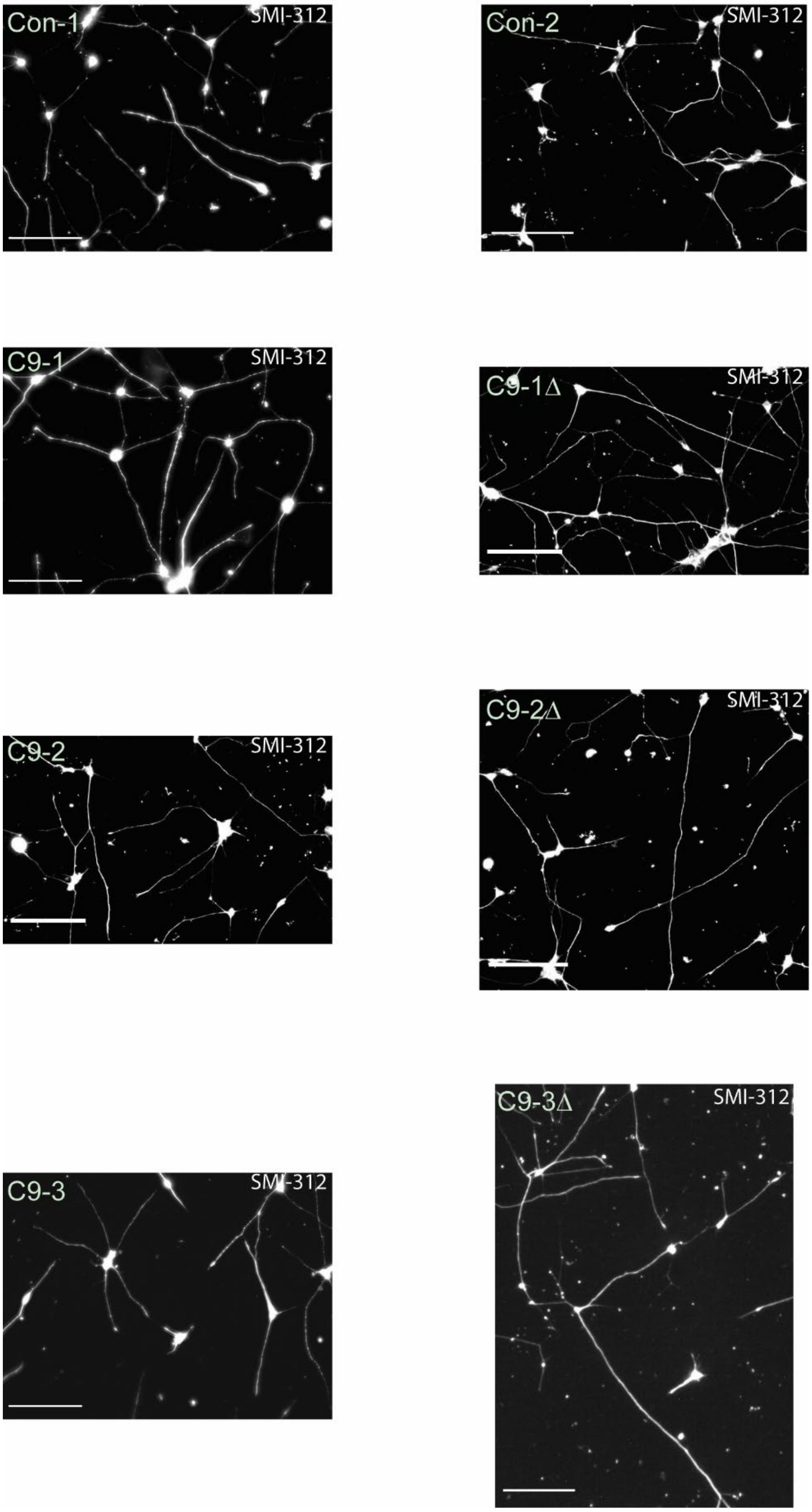

Supplementary Fig 1: Motor neuron neurite length. Representative images of human induced pluripotent stem cell derived spinal motor neurons depicting axonal staining via SMI-312, 7 days post platedown. Two independent controls (Con-1, Con-2) and three *C9orf72* mutant-isogene pairs (C9-1, C9-1Δ; C9-2, C9-2Δ; C9-3, C9-3Δ) are shown. Scale bars = 100 µm.

Supplementary Fig 2

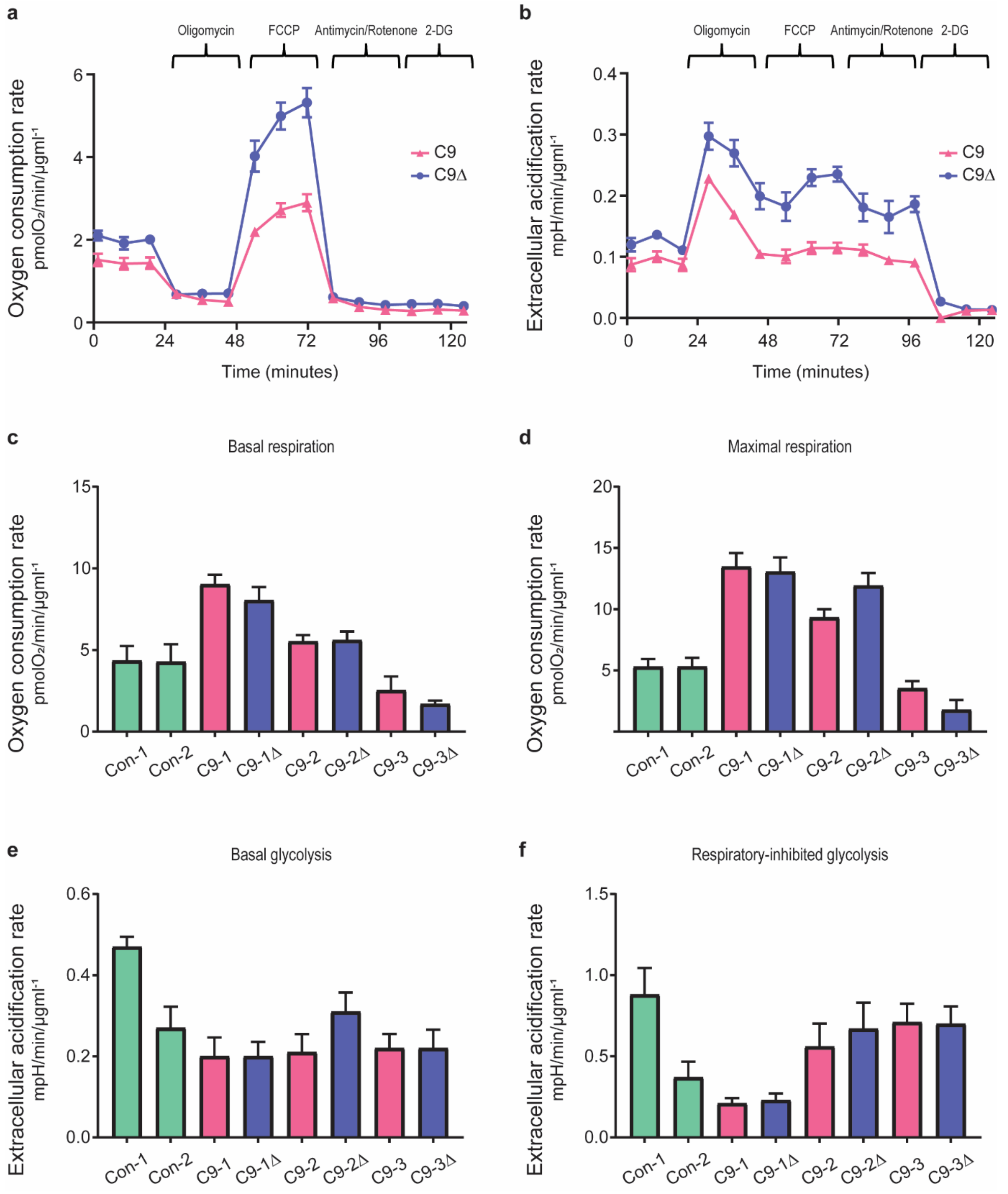

Supplementary Fig 2: Metabolic profiling using Seahorse Analyzer. **a**, Representative Seahorse Analyzer mitochondrial stress test trace showing oxygen consumption rate (OCR) measurements, normalised to total protein, following sequential compound injection into the cell microculture plate of: oligomycin, FCCP, antimycin/rotenone, and 2-deoxy-d-glucose, as shown, for a mutant-isogene *C9orf72* pair. **b**, Representative Seahorse Analyzer mitochondrial stress test trace showing extracellular

acidification rate (ECAR) measurements, normalised to total protein, following sequential compound injection into the cell microculture plate of: oligomycin, FCCP, antimycin/rotenone, and 2-deoxy-d-glucose, as shown, for a mutant-isogene *C9orf72* pair. **c-d**, Quantification of OCR as measured by the Seahorse Analyzer, normalised to the amount of total protein, denoting basal (**c**) and maximal FCCP-uncoupled (**d**) mitochondrial respiration of *C9orf72*-iPSCs and isogenic paired controls; two independent healthy controls are also shown. Data are represented as mean ± S.E.M; $n \geq 4$ wells per line per experiment, with experiments repeated in three separately passaged cultures ($N = 3$). Statistical significance was evaluated with one-way ANOVA. **e-f**, Quantification of the glycolytic capacity as estimated by ECAR measurements via the Seahorse Analyzer, normalised to the amount of total protein, denoting basal (**e**) and respiratory-inhibited (**f**) glycolysis of *C9orf72*-MNs and isogenic paired controls; two independent healthy controls are also shown. Data are represented as mean ± S.E.M; $n \geq 4$ wells per line per experiment, with experiments repeated in three independent cultures from different differentiations ($N = 3$). Statistical significance was evaluated with one-way ANOVA.

Supplementary Fig 3

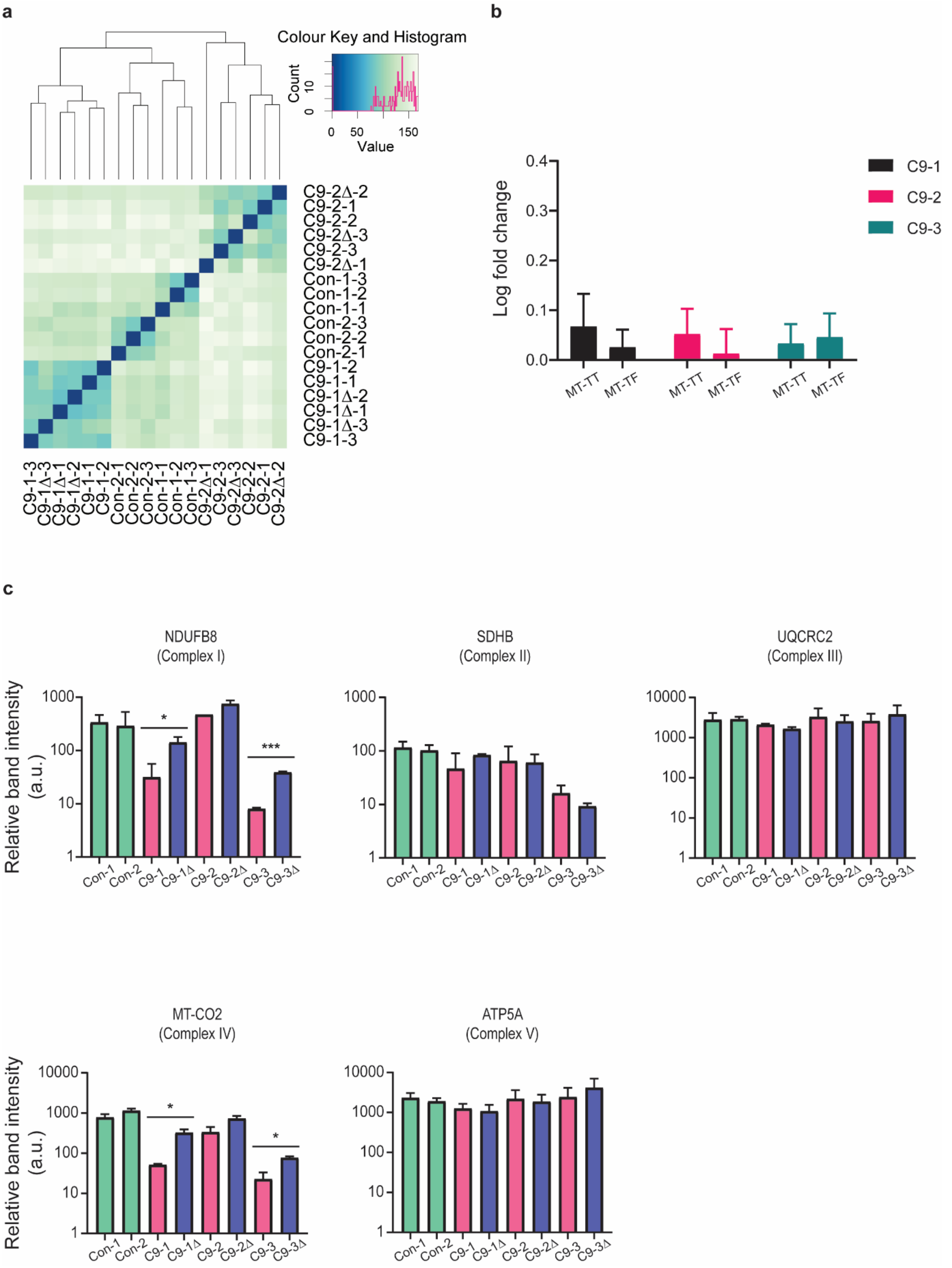

Supplementary Fig 3: Supplementary RNA-seq, RT-PCR and western blot quantification data. **a**, Heat map and dendrogram depicting unsupervised hierarchical clustering of RNA sequencing reads

from MN samples derived from 3 independent differentiations of 2 independent controls [Con-1, Con-2], 2 independent patient-derived *C9orf72* lines [C9-1, C9-2] with their corresponding isogenic controls [C9-1Δ, C9-2Δ]. **b**, Log_{10} mean fold change ± S.E.M. of gene expression normalised to the respective isogenic control determined via RT-PCR for C9-1 pair (black bars), C9-2 pair (red bars), and C9-3 pair (green bars) for mitochondrial t-RNA transcripts: MT-TT and MT-TF. **c**, Densitometric analysis of Western blot data from 3 independent patient-derived *C9orf72* lines [C9-1, C9-2, C9-3] with their corresponding isogenic controls [C9-1Δ, C9-2Δ, C9-3Δ], and two independent controls. Data plotted for electron transport chain complex I (NDUFB8 subunit), complex II (SDHB subunit), complex III (UQCRC2), complex IV (MT-CO2) and complex V (ATP5A) as mean intensity ± S.E.M (a.u., arbitrary units), normalised to GAPDH loading control, from three independent cultures from different differentiations ($N = 3$). $^{*}p < 0.05$, $^{**}p < 0.01$, $^{***}p < 0.001$.

Supplementary Fig 4

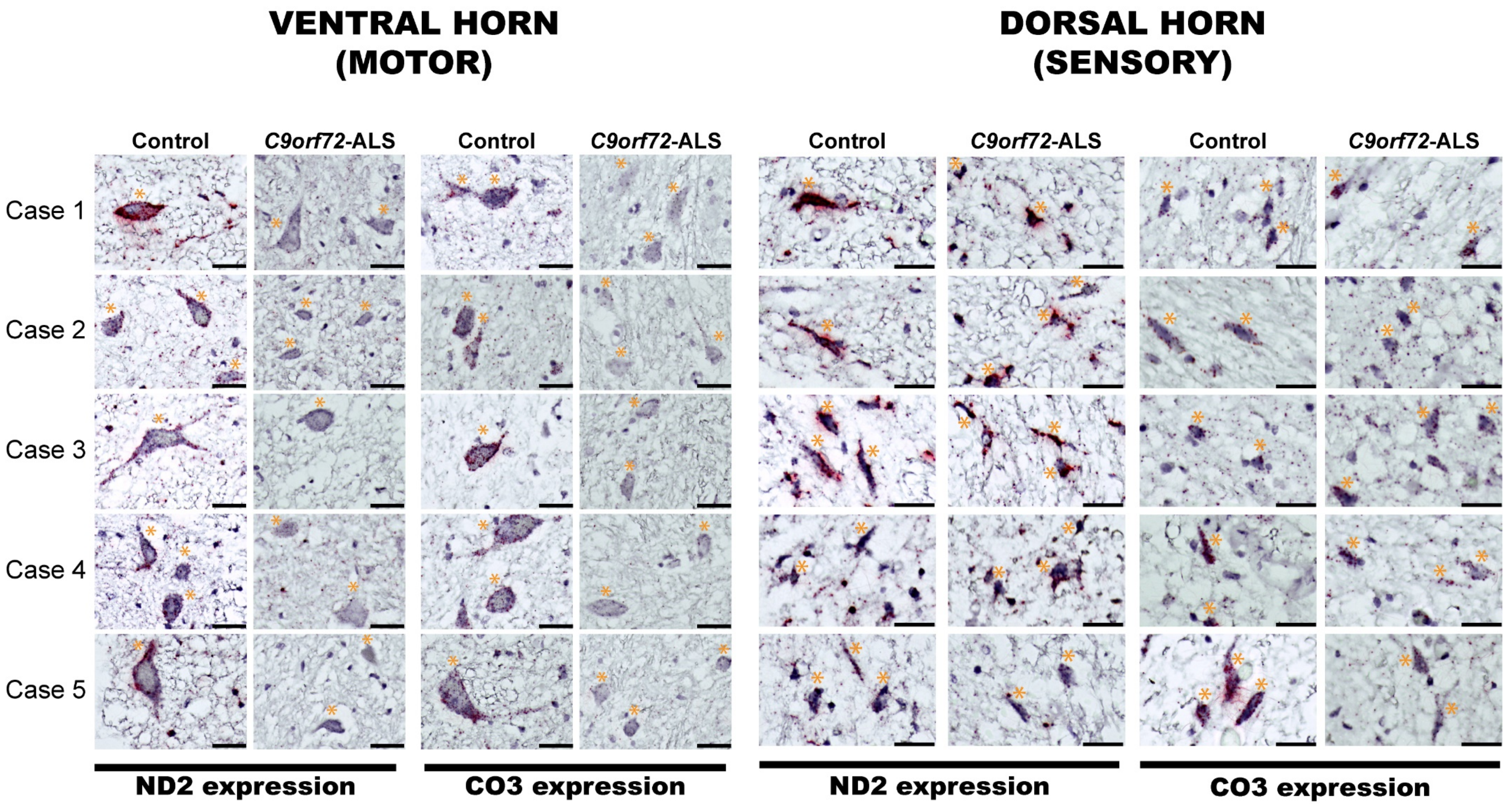

Supplementary Fig 4: **Examination of human post-mortem ventral horn spinal motor, *versus* dorsal horn spinal sensory, neurons in *C9orf72* cases *versus* controls shows motor neuron selective reduction in gene expression of complexes I and IV of the mitochondrial electron transport chain.** Photomicrographs showing reduced expression of MT-ND2 and MT-CO3 transcripts in ventral horn motor neurons (left), but not in dorsal horn spinal sensory neurons (right), from post-mortem spinal cord tissue derived from each of 5 individuals with a *C9orf72* hexanucleotide repeat expansion mutation compared with their age- and sex-matched control case. Red dots highlight individual mRNA molecules of MT-ND2 or MT-CO3. Tissue is counterstained with haematoxylin. Scale bars = 50 µm. Orange asterisk denotes motor (ventral horn) or sensory (dorsal horn) neurons.

Supplementary Fig 5

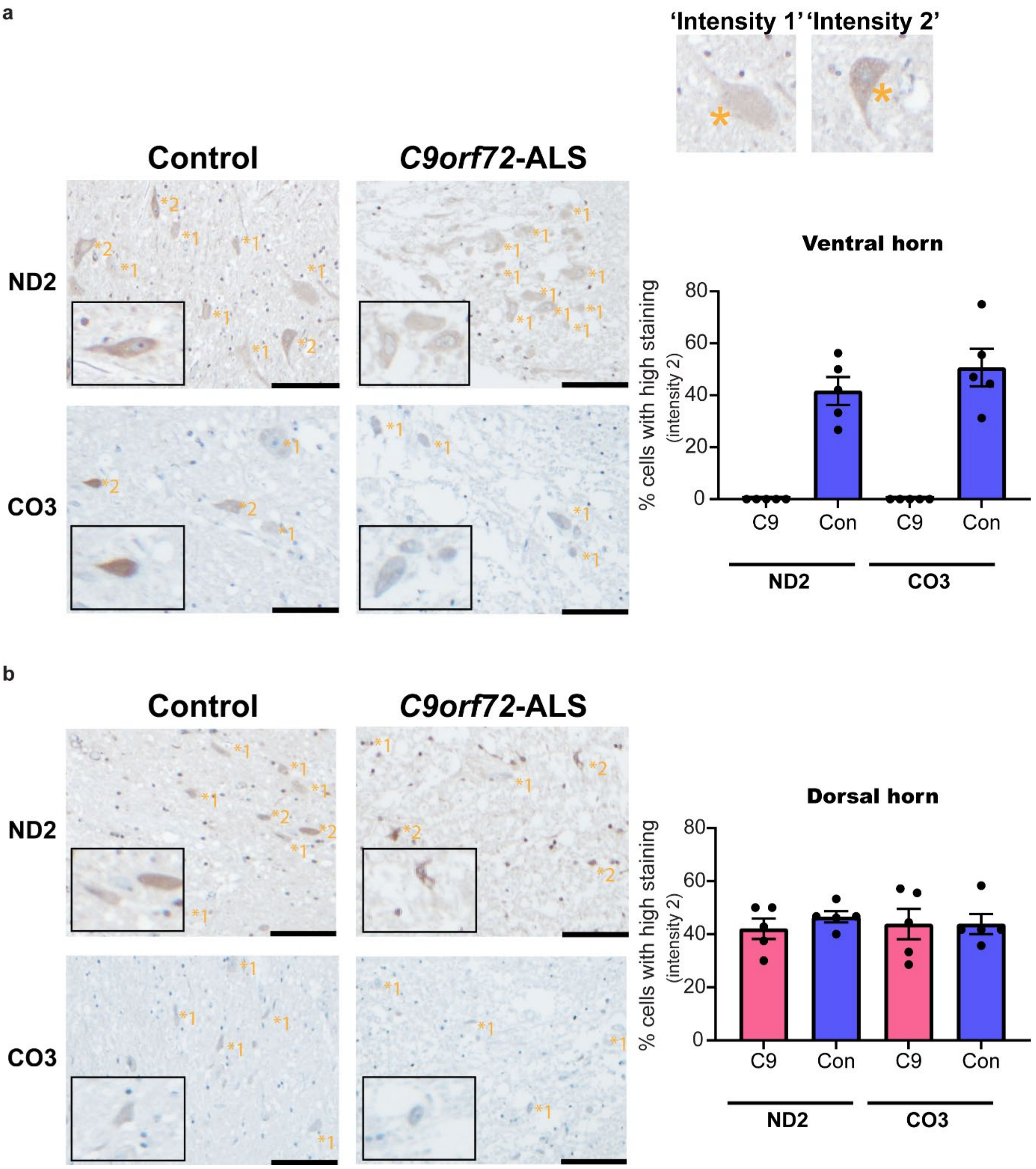

Supplementary Fig 5: **Reduced levels of MT-ND2 and MT-CO3 protein in human post-mortem ventral horn spinal motor, *versus* dorsal horn spinal sensory, neurons in *C9orf72* cases *versus* controls.** Representative photomicrographs taken at 20x magnification, with inset showing 40x magnification. Sections show immunohistochemical staining for ND2 and CO3 protein (detected using DAB chromogen), counterstained with haematoxylin, in ventral (**a**) and dorsal (**b**) horns of the spinal cord. Orange asterisk indicates a motor neuron in the ventral sections and a sensory neuron in the dorsal sections, and the adjacent numbers (either '1' or '2') indicate the staining intensity for that cell, based

on the key, as displayed in the top right corner of the figure (see also Material and Methods). Scale bars = 120 µm. Bar charts show the quantification of the percentage of cells with intensity '2' (high staining intensity) for 5 *C9orf72* cases *versus* age- and sex-matched controls, evaluating 10 cells per case/control. There is significant reduction in immunoreactivity observed in ventral motor neurons in *C9orf72* for both ND2 and CO3 mitochondrial proteins ($p < 0.0001$), whereas there is no difference in immunoreactivity in the dorsal sensory neurons when comparing cases *versus* controls (see Supplementary Table 3 for accompanying statistical significance calculations).

Supplementary Fig 6

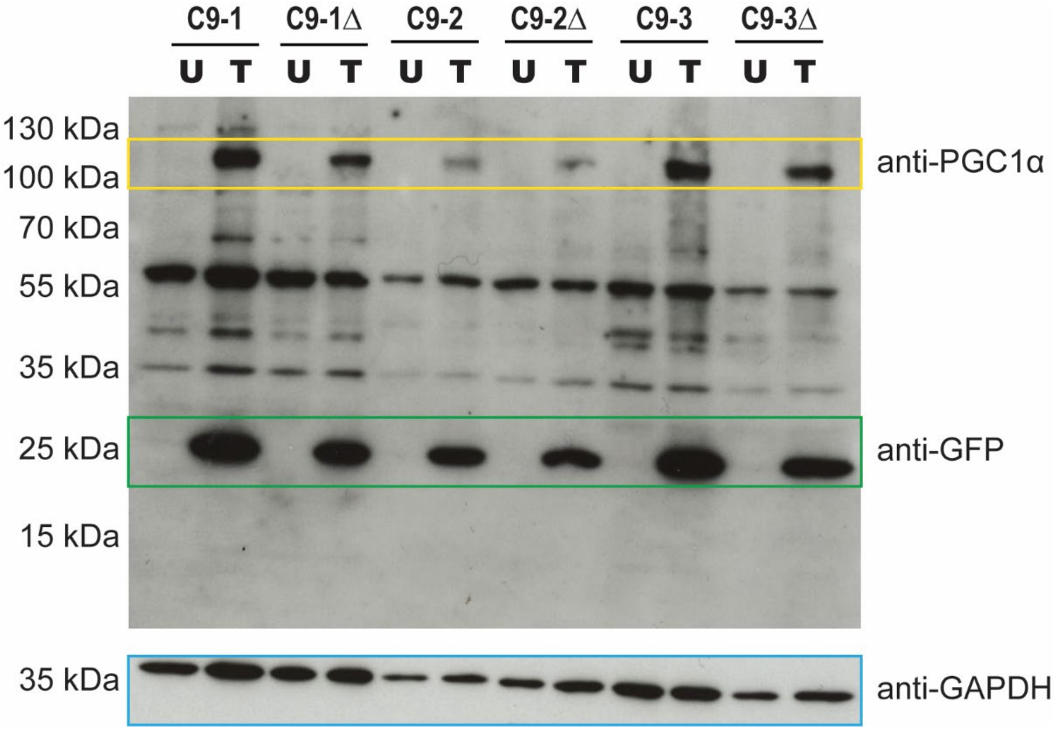

Supplementary Fig 6: **Lentiviral mediated transduction of PGC1α-P2A-eGFP.** Western blot following 4-20% gradient Tris-glycine gel electrophoresis showing untransduced (U) and transduced (T) MN protein lysates. Specific bands for PGC1α (~120 kDa), GFP (~27 kDa), and GAPDH (~36 kDa) loading control are denoted by yellow, green and blue boxes, respectively.

Supplementary Table 1: Primer Details.

Gene name	HGNC ID	Primer sequence (5' – 3')	Product size (bp)	Annealing temperature (°C)
MT-TT	HGNC:7499	Forward AAACTAATACACCAGTCTTGT Reverse AAGGTTTTCATCTCCGGTTT	41	57
MT-TF	HGNC:7481	Forward ACCTCCTCAAAGCAATACACTG Reverse GGTGATGTGAGCCCGTCTAA	49	57
18S rRNA	HGNC:44278	Forward GTAACCCGTTGAACCCCATT Reverse CCATCCAATCGGTAGTAGCG	151	57
B2M	HGNC:914	Forward CCAGCAGAGAATGGAAAGTCAA Reverse TCTCTCTCCATTCTTCAGTAAGTCAACT	96	64
MT-ND1	HGNC:7455	Forward CCCTAAAACCCGCCACATCT Reverse GAGCGATGGTGAGAGCTAAGGT	69	64
MT-ND2	HGNC:7456	Forward CTTCTGAGTCCCAGAGGTTA Reverse GTGAGGGAGAGATTTGGTATATG	110	57
MT-ND4	HGNC:7459	Forward CTCACTGCCCAAGAACTATC Reverse CATAAGTGGAGTCCGTAAAGAG	104	57
MT-CO2	HGNC:7421	Forward CCCTCCCTTACCATCAAATC Reverse TCGCCTGGTTCTAGGAATA	119	57
MT-CO3	HGNC:7422	Forward CTCGCATCAGGAGTATCAATC Reverse AGGCTTGTAGGAGGGTAAA	124	57
MT-ATP6	HGNC:7414	Forward CCCACCTCCAAATATCTCATC Reverse GATCAGGTTCGTCCTTTAGTG	113	57

Supplementary Table 2: Age- and sex-matched controls used in post-mortem work.

Abbreviations: F – Female; M – Male.

Control ID (Sex)	Age at death (years)
1 (F)	64
2 (M)	52
3 (F)	62
4 (F)	64
5 (M)	59

Supplementary Table 3: Statistical significance calculations for data presented in Supplementary Fig 5.

Ventral horn immunohistochemistry:

**ND2 contingency table**	Intensity 1	Intensity 2	**Total**
Control	58	42	100
Cases	100	0	100
**Total**	158	42	200

Chi-squared (with Yates's correction) equals 50.663 with 1 degrees of freedom.
The two-tailed *p* value is less than 0.0001.

**CO3 contingency table**	Intensity 1	Intensity 2	**Total**
Controls	49	51	100
Cases	100	0	100
**Total**	149	51	200

Chi-squared (with Yates's correction) equals 65.798 with 1 degrees of freedom.
The two-tailed *p* value is less than 0.0001.

Dorsal horn immunohistochemistry:

**ND2 contingency table**	Intensity 1	Intensity 2	**Total**
Controls	53	47	100
Cases	58	42	100
**Total**	111	89	200

Chi-squared equals 0.324 with 1 degrees of freedom.
The two-tailed *p* value equals 0.5693.

**CO3 contingency table**	Intensity 1	Intensity 2	**Total**
Controls	56	44	100
Cases	56	44	100
**Total**	112	88	200

Chi-squared equals 0.000 with 1 degrees of freedom.
The two-tailed *p* value equals 1.0000.

Supplementary Data 1: Spreadsheet of bioinformatics data. Sheets 1 and 2 show the significantly up- and down-regulated genes in *C9orf72* MNs, respectively; sheets 3, 4 and 5 show the results of GO analysis for biological process, cellular compartment, and molecular function, respectively; sheet 6 shows the results of promoter analysis.

Supplementary Video 1: Axonal mitochondrial motility. Time-lapse videos (at 7 frames per second) of a ~100 µm stretch of axon from a *C9orf72* MN (**a**) and its paired isogenic control MN (**b**) showing DsRed2 labelled mitochondria, and their lack of movement in *C9orf72* relative to its isogenic control.

Supplementary Video 2: Axonal mitochondrial motility after PGC1α overexpression. Time-lapse videos (at 7 frames per second) of a ~100 µm stretch of axon from a *C9orf72* MN (**a**) and its paired isogenic control MN (**b**) showing DsRed2 labelled mitochondria, after overexpression of PGC1α. PGC1α is shown to boost mitochondrial motility and number in both the *C9orf72* MN and its isogenic control.
